# Specific Blood RNA Profiles in Individuals with Acute Spinal Cord Injury as Compared with Trauma Controls

**DOI:** 10.1155/2023/1485135

**Published:** 2023-01-12

**Authors:** Qing Chen, Longqing Wang, Hao Wu, Cheng Ye, Dong Xie, Qi Zhao, Qi Zhu, Chenhui Xu, Lili Yang

**Affiliations:** ^1^Spine Center, Department of Orthopaedics, Shanghai Changzheng Hospital, Second Affiliated Hospital of Naval Medical University, Shanghai, China; ^2^Department of Orthopaedics, No. 905 Hospital of PLA Navy, Shanghai, China

## Abstract

**Background:**

Spinal cord injury (SCI) is known to cause a more robust systemic inflammatory response than general trauma without CNS injury, inducing severe secondary organ damage, especially the lung and liver. Related studies are principally focused on the mechanisms underlying repair and regeneration in the injured spinal cord tissue. However, the specific mechanism of secondary injury after acute SCI is widely overlooked, compared with general trauma.

**Methods:**

Two datasets of GSE151371 and GSE45376 related to the blood samples and spinal cord after acute SCI were selected to identify the differentially expressed genes (DEGs). In GSE151371, functional enrichment analysis on specific DEGs of blood samples was performed. And the top 15 specific hub genes were identified from intersectional genes between the specific upregulated DEGs of blood samples in GSE151371 and the upregulated DEGs of the spinal cord in GSE45376. The specific functional enrichment analysis and the drug candidates of the hub genes and the miRNAs-targeted hub genes were also analyzed and predicted.

**Results:**

DEGs were identified, and a total of 64 specific genes were the intersection of upregulated genes of the spinal cord in GSE45376 and upregulated genes of human blood samples in GSE151371. The top 15 hub genes including HP, LCN2, DLGAP5, CEP55, HMMR, CDKN3, PRTN3, SKA3, MPO, LTF, CDC25C, MMP9, NEIL3, NUSAP1, and CD163 were calculated from the 64 specific genes. Functional enrichment analysis of the top 15 hub genes revealed inflammation-related pathways. The predicted miRNAs-targeted hub genes and drug candidates of hub genes were also performed to put forward reasonable treatment strategies.

**Conclusion:**

The specific hub genes of acute SCI as compared with trauma without CNS injury were identified. The functional enrichment analysis of hub genes showed a specific immune response. Several predicted drugs of hub genes were also obtained. The hub genes and the predicted miRNAs may be potential biomarkers and therapeutic targets and require further validation.

## 1. Introduction

As a traumatic disease caused by various traffic accidents and violent injuries, spinal cord injury (SCI) mainly inflicts the structure and function of the spinal cord directly with negative effects on the physical and mental health of patients and huge economic and social burdens [1–3]. Several severe secondary injuries including inflammation, oxidative stress, and apoptosis may ensue after SCI [1, 4, 5]. In patients suffering traumatic diseases without damage to the central nervous system (CNS), a secondary inflammatory process may be triggered, which can last for weeks and involves several inflammatory mediators [6]. Several studies have suggested that CNS injury would lead to a more robust systemic inflammatory reaction than traumatic diseases without CNS injury, with injury to the lungs and liver [7–11].

RNA sequencing (RNA-seq) has been extensively used to elucidate the molecular mechanisms and hub genes of animals with SCI by comprehensive analysis [12, 13]. However, the specific mechanism mediating the more intense systemic inflammation response in patients after SCI exists uncertain and needs more studies [7, 14]. Many kinds of research on identifying cerebrospinal fluid (CSF) and serum biomarkers through proteomics and RNAs after SCI were restricted to the measurement and hard to degrade [15–20]. In this context, a suitable approach is needed to identify the specific molecular changes in patients after SCI. The “sensors” of SCI-induced molecules were performed by circulating immune cells [15]. Peripheral white blood cells (WBC) transcriptome offer a significant source of peripheral immune response to the signals of SCI in the body [15].

In our study, two RNA-seq datasets of GSE151371 related to blood samples and GSE45376 related to the spinal cord of acute SCI were collected from Gene Expression Omnibus (GEO) database [15, 21]. A detailed bioinformatic analysis was conducted to unravel the specific mechanisms of acute SCI as compared with traumatic diseases without CNS injury. The identified specific hub genes and pathways are critical for improvements in prognosis and therapeutic strategies for acute SCI.

## 2. Materials and Methods

### 2.1. Data Sources

**Figure 1:**
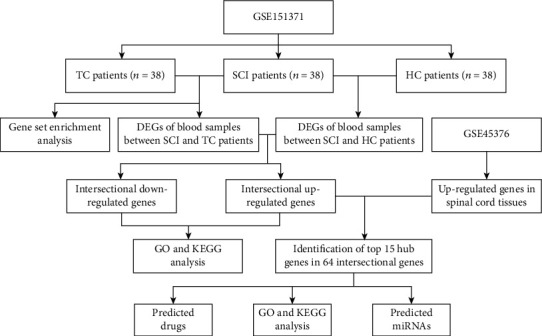
The general flow diagram of this study.

The RNA-seq datasets of GSE151371 and GSE45376 were downloaded from the GEO database (https://www.ncbi.nlm.nih.gov/geo/). The GSE151371 dataset composes of 58 acute blood samples of humans divided into 3 groups, including 10 healthy uninjured controls (HC), 10 trauma controls without central nervous system injuries (TC), and 38 patients with SCI. In the GSE45376 dataset, 3 mice with acute SCI and 2 mice who received only a laminectomy with non-SCI were selected to further analysis. The general flow chart of this study is summarized in Figure 1.

### 2.2. Gene Set Enrichment Analysis

The packages of “GSEABase” and “clusterProfiler” in R (version 4.1.2) were used to support gene set enrichment analysis (GSEA) between the groups of SCI and TC in datasets of GSE151371. The annotated gene set of “c2.cp.kegg.v7.1.symbols.gmt” was obtained from the MSigDB (https://www.gsea-msigdb.org/gsea/msigdb/). The gene sets were selected to satisfy the conditions of normalized enrichment score (NES) >1.5, *P* value < 0.05, and *P* value cutoff = 1.

### 2.3. Differentially Expressed Genes

The package of “limma” and “DESeq2” in R was chosen to analyze differentially expressed genes (DEGs) between groups of HC and SCI, TC and SCI, and the selected mice groups, under the conditions of |log2FoldChange| > 1 and false discovery rate (FDR) value <0.05. The DEGs were used for volcano plotting and taking the intersection.

### 2.4. Functional Enrichment Analysis of Intersectional DEGs

The Venn diagram was used to show the intersectional genes of upregulated and downregulated DEGs between HC and SCI and TC and SCI through the online tool of jvenn (http://www.bioinformatics.com.cn/static/others/jvenn/), respectively [22]. The packages of “GOplot” and “clusterProfiler” in R were used to identify the top 10 Gene Ontology (GO) biological processes and the Kyoto Encyclopedia of Genes and Genomes (KEGG) pathway of 64 intersectional upregulated and downregulated DEGs.

### 2.5. Identification of Upregulated Hub Genes between Two Datasets

The online tool jvenn was performed to calculate the intersection between upregulated DEGs in the mice groups and the intersectional upregulated genes mentioned in the above section [22]. Protein-protein interaction (PPI) networks of the intersectional genes were identified through STRING's website (https://string-db.org/), under the condition of a confidence score >0.4. The plug-in unit of cytoHubba in Cytoscape software (version 3.8.2) was utilized to identify the top 15 hub genes in the PPI network. The analysis of GO biological processes and the KEGG pathways of the hub genes was performed with the same method.

### 2.6. Identification of Drug Candidates and Predicted miRNAs

The online tool Enrichr (https://maayanlab.cloud/Enrichr/) was used to identify predicted miRNAs-targeted hub genes and the drug candidates of hub genes. The predicted miRNAs were selected through miRTarBase_2017 of Enrichr. The drug candidates were obtained through the DSigDB of Enrichr. The identified terms are ranked from high to low based on a combined score.

## 3. Results

### 3.1. DEGs between SCI and Control Groups

**Figure 2:**
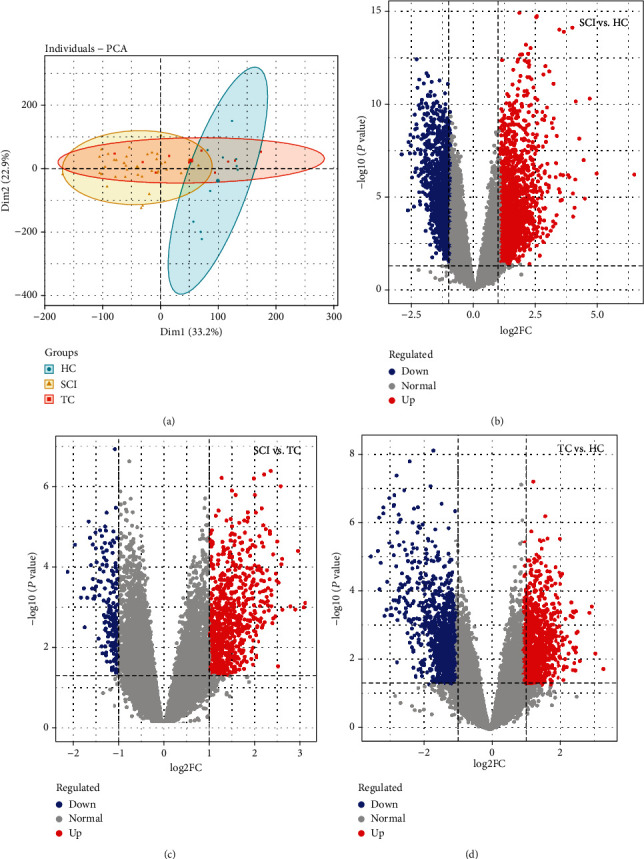
PCA and volcano plots of GSE151371. (a) The PCA plot shows the dissimilarity between SCI, TC, and HC groups. Volcano plot visualization of DEGs between (b) SCI and HC groups, (c) SCI and TC groups, and (d) TC and HC groups, respectively. PCA: principal component analysis; SCI: spinal cord injury; TC: trauma controls without central nervous system injuries; HC: healthy uninjured controls; DEGs: differentially expressed genes.

**Figure 3:**
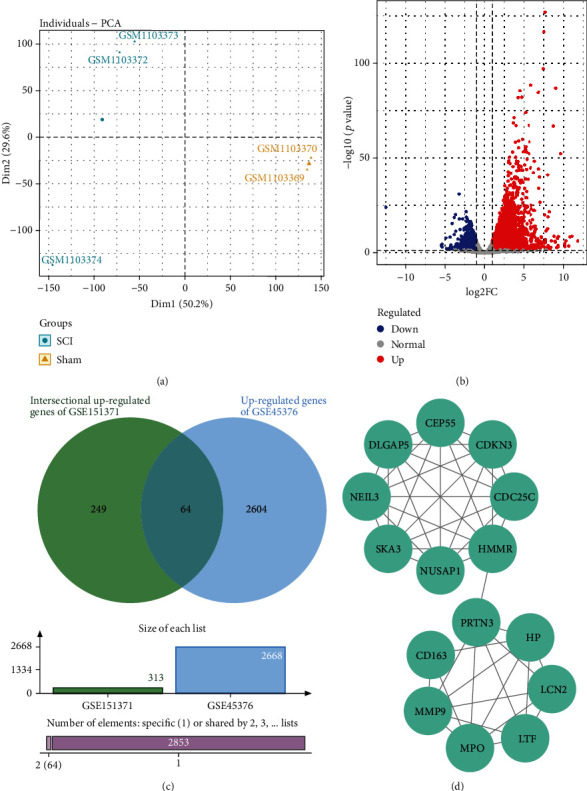
PCA and volcano plots of GSE45376 and the PPI network of hub genes. (a) The PCA plot shows the dissimilarity between SCI and sham groups. (b) Volcano plot visualization of DEGs between SCI and sham groups. (c) The 64 intersectional upregulated genes between the 2668 upregulated genes of the spinal cord in GSE45376 and the 303 intersectional upregulated genes in GSE151371. (d) PPI network of the top 15 hub genes identified from the 64 intersectional upregulated genes. PCA: principal component analysis; SCI: spinal cord injury; PPI: protein-protein interaction; DEGs: differentially expressed genes.

A principal component analysis (PCA) plot of the selected groups in two datasets revealed what appear to be diverse groupings (Figures 2(a) and 3(a)). A total of 792 upregulated and 210 downregulated genes were found in the blood samples between the SCI and TC groups. 1733 upregulated and 1459 downregulated genes were found in blood samples between the SCI and HC groups. 1157 upregulated and 1080 downregulated genes were found in blood samples between the TC and HC groups. 2668 upregulated and 1123 downregulated genes were found in the spinal cord between SCI and sham groups in mice. DEGs among groups of the datasets mentioned above are described in the supplementary file (available here). Volcano plots in Figures 2(b)–2(d) demonstrated the DEGs between groups above in the GSE151371 dataset. Volcano plots in Figure 3(b) demonstrated the DEGs between groups above in the GSE45376 dataset.

### 3.2. GSEA in Patients with SCI

**Figure 4:**
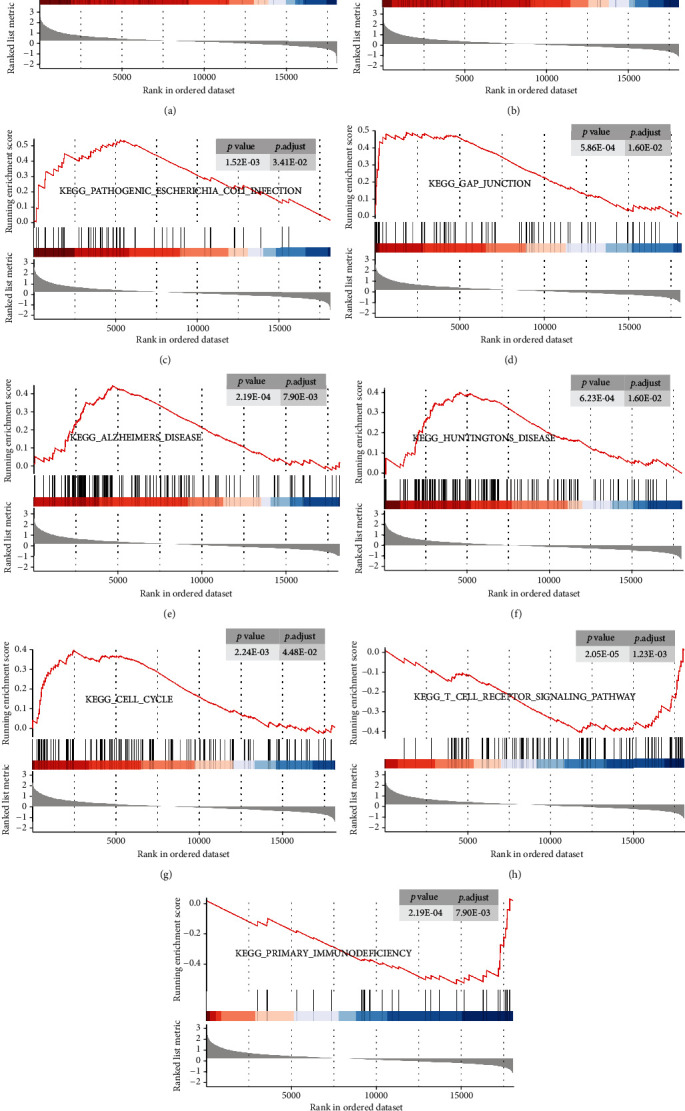
GSEA of specific pathways in human blood samples after SCI as compared with TC. (a–g) Seven representative gene sets enriched in human blood samples with SCI. (h, i) Two representative gene sets inhibited in human blood samples with SCI. GSEA: gene set enrichment analysis; SCI: spinal cord injury; TC: trauma controls without central nervous system injuries.

GSEA was performed to map the KEGG pathways between SCI and TC groups in the GSE151371 dataset. Nine significant pathways were displayed (Table 1; Figures 4(a)–4(i)). GSEA showed that many KEGG pathways including Parkinson's disease, oxidative phosphorylation, pathogenic *Escherichia coli* infection, gap junction, Alzheimer's disease, Huntington's disease, and cell cycle were enriched in SCI in patients. T cell receptor signaling pathway and primary immunodeficiency gene sets were inhibited in patients with SCI.

### 3.3. GO Biological Processes and KEGG Pathways of Intersectional Genes

**Figure 5:**
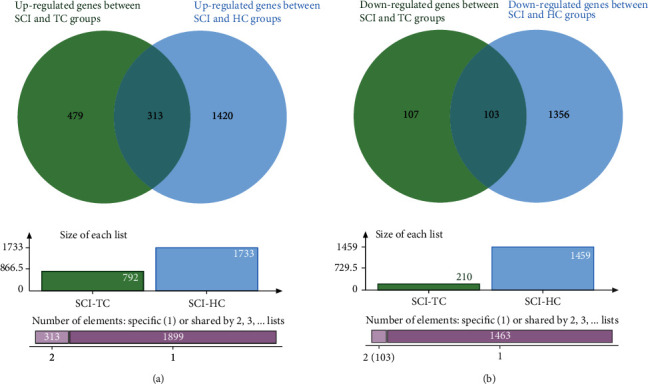
Venn plots of the intersectional DEGs in GSE151371. (a) 313 intersectional upregulated genes were identified between the upregulated DEGs of SCI and TC groups and SCI and HC groups. (b) 103 intersectional downregulated genes were identified between the downregulated DEGs of SCI and TC groups and SCI and HC groups. SCI: spinal cord injury; TC: trauma controls without central nervous system injuries; HC: healthy uninjured controls; DEGs: differentially expressed genes.

**Figure 6:**
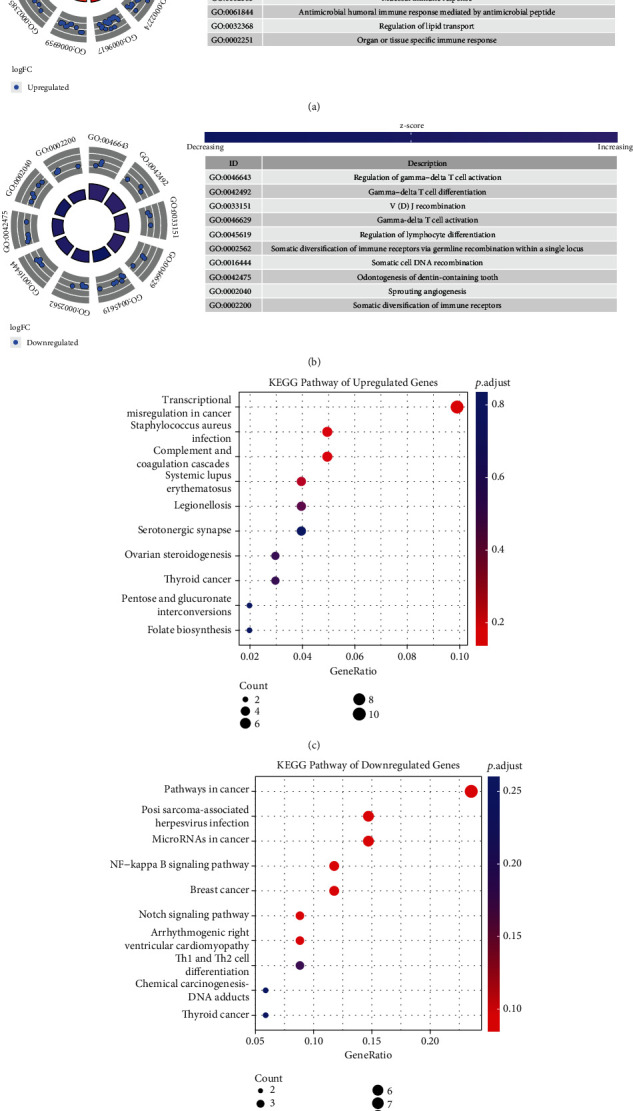
GO biological processes and KEGG pathways of the intersectional genes. (a, b) GO biological processes of the 313 intersectional upregulated genes. (c, d) KEGG pathways of the 103 intersectional downregulated genes. GO: Gene Ontology; KEGG: Kyoto Encyclopedia of Genes and Genomes.

A total of 313 intersectional upregulated genes and 103 downregulated genes were identified between DEGs of SCI and TC groups and SCI and HC groups (Figure 5). The top 10 GO biological processes and KEGG pathways are illustrated in Figure 6. The GO biological processes of intersectional genes are shown in Figures 6(a) and 6(b). The upregulated genes were mainly enriched in defense response to fungus, defense response to bacterium, response to fungus, myeloid leukocyte activation, response to bacterium, humoral immune response, mucosal immune response, antimicrobial humoral immune response mediated by antimicrobial peptide, regulation of lipid transport, and organ or tissue specific immune response. The downregulated genes were mainly enriched in the regulation of gamma-delta T cell activation, gamma-delta T cell differentiation, V(D)J recombination, gamma-delta T cell activation, regulation of lymphocyte differentiation, somatic diversification of immune receptors via germline recombination within a single locus, somatic cell DNA recombination, odontogenesis of dentin-containing tooth, sprouting angiogenesis, and somatic diversification of immune receptors.

The KEGG pathways of intersectional genes are shown in Figure 6(c)–6(d). The upregulated genes were mainly focused on transcriptional misregulation in cancer, staphylococcus aureus infection, complement and coagulation cascades, systemic lupus erythematosus, legionellosis, ovarian steroidogenesis, thyroid cancer, pentose and glucuronate interconversions, serotonergic synapse, and folate biosynthesis. The downregulated genes were mainly enriched in NF-kappa B (NF-*κ*B) signaling pathway, Kaposi sarcoma-associated herpesvirus infection, pathways in cancer, Notch signaling pathway, microRNAs in cancer, breast cancer, arrhythmogenic right ventricular cardiomyopathy, Th1 and Th2 cell differentiation, chemical carcinogenesis-DNA adducts, and thyroid cancer.

### 3.4. Identification and Functional Enrichment Analysis of Hub Genes

A total of 64 intersectional upregulated genes were analyzed between upregulated genes of GSE45376 and 313 intersectional upregulated genes mentioned above (Figure 3(a)). The top 15 hub genes were identified in the PPI networks of the 64 genes through cytoHubba (Table 2; Figures 3(c) and 3(d)).

**Figure 7:**
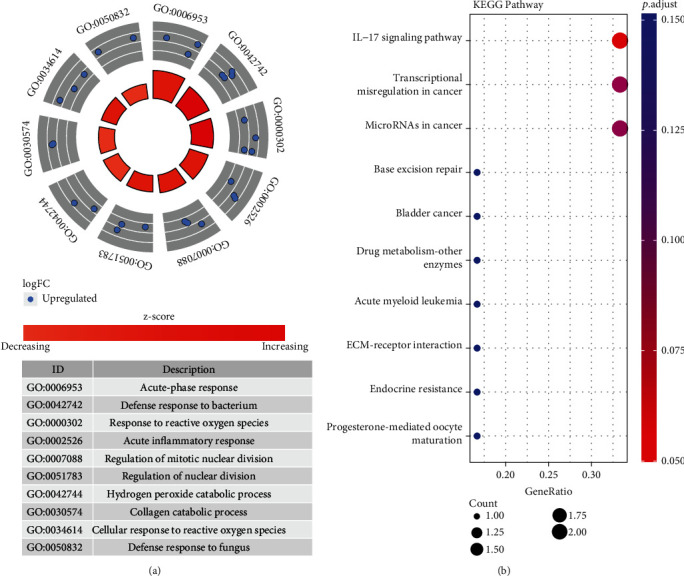
GO biological processes and KEGG pathways of top 15 hub genes. (a) GO biological processes of hub genes. (b) KEGG pathways of the hub genes. GO: Gene Ontology; KEGG: Kyoto Encyclopedia of Genes and Genomes.

In GO biological processes, the hub genes were mainly clustered in acute-phase response, defense response to bacterium, response to reactive oxygen species, acute inflammatory response, regulation of mitotic nuclear division, regulation of nuclear division, hydrogen peroxide catabolic process, collagen catabolic process, cellular response to reactive oxygen species, and defense response to fungus (Figure 7(a)). In KEGG pathways, the hub genes were mainly enriched in the IL-17 signaling pathway, transcriptional misregulation in cancer, microRNAs in cancer, base excision repair, bladder cancer, drug metabolism-other enzymes, acute myeloid leukemia, ECM-receptor interaction, endocrine resistance, and progesterone-mediated oocyte maturation (Figure 7(b)).

### 3.5. Identification of Predicted miRNAs

The predicted miRNAs-targeted hub genes were further investigated to reveal the specific noncoding RNAs of SCI. The top 10 predicted miRNAs were obtained according to the combined score (Table 3). The hsa-miR-4750, hsa-miR-3146, hsa-miR-671-3p, hsa-miR-517a, hsa-miR-517c, hsa-miR-4757-3p, hsa-miR-621, hsa-miR-887, hsa-miR-4259, and hsa-miR-95 were identified by the hub genes.

### 3.6. Identification of Drug Candidates

This study went further to search for the potential drug for the treatment of SCI. The top 10 drug candidates were obtained according to the combined score (Table 4). Dioxocerium, abacavir, potassium persulfate, cryptolepine, lucanthone, oxozinc, 6401-97-4, butein, pimaric acid, and 4,4′-methylenebis(2-chloroaniline) were identified by the hub genes.

## 4. Discussion

SCI is a kind of devastating disease worldwide with a high incidence, disability, and mortality rate [1, 23]. Several studies have shown that unique molecular features of SCI were identified when compared with traumatic diseases without CNS injury [7, 14]. Specifically, more intense system inflammatory reactions and severe damage to the liver and lungs were reported in animal and human studies [7–9, 14]. However, specific molecular mechanisms are still unclear and need to be further studied. In this study, the specific hub genes and pathways were confirmed through bioinformatic analysis of the acute SCI-related RNA-seq datasets.

The RNA-seq datasets of GSE151371 and GSE45376 were analyzed in R software. The PCA shows clear differences between groups in the two datasets.

In dataset GSE151371, 792 upregulated and 210 downregulated genes were found between the SCI and TC groups, and 1733 upregulated and 1459 downregulated genes were found between the SCI and HC groups. In dataset GSE45376, 2803 upregulated and 1314 downregulated genes were found between SCI and sham groups. GSEA was used to perform an unbiased analysis between the SCI and TC groups with blood samples of humans in GSE151371. The results of GSEA indicated that SCI activated the oxidative phosphorylation and cell cycle and inhibited the T cell receptor signaling pathway and primary immunodeficiency. The intersectional DEGs between SCI and TC groups and SCI and HC groups in GSE151371 were calculated, respectively. Subsequently, the top 10 terms of GO biological processes and KEGG analysis were performed among the intersectional DEGs. Among the 313 intersectional upregulated genes, GO biological processes revealed some specific mechanisms of the immune response, including humoral immune response, mucosal immune response, and organ or issue-specific immune response. The KEGG pathways revealed complement and coagulation cascades and pentose and glucuronate interconversions. While among the 103 intersectional downregulated genes, GO biological processes showed several specific mechanisms of changes related to the functional state of the cells, including regulation of gamma-delta T cell activation, gamma-delta T cell differentiation, gamma-delta T cell activation, regulation of lymphocyte differentiation, somatic diversification of immune receptors via germline recombination within a single locus, somatic cell DNA recombination, and somatic diversification of immune receptors. The KEGG pathways showed NF-*κ*B signaling pathway, Notch signaling pathway, and Th1 and Th2 cell differentiation. These results shed light on specific mechanisms by which SCI leads to abnormal immune function in patients.

A total of 64 specific genes were identified between the 313 upregulated genes of the spinal cord in GSE45376 and the 2668 intersectional upregulated genes of human blood samples mentioned above. The top 15 hub genes, including haptoglobin (HP), lipocalin-2 (LCN2), DLG-associated protein 5 (DLGAP5), centrosomal protein 55 (CEP55), hyaluronan-mediated motility receptor (HMMR), cyclin-dependent kinase inhibitor 3 (CDKN3), proteinase 3 (PRTN3), spindle and kinetochore-associated complex subunit 3 (SKA3), myeloperoxidase (MPO), lactotransferrin (LTF), cell division cycle 25C (CDC25C), matrix metallopeptidase 9 (MMP9), Nei-like DNA glycosylase 3 (NEIL3), nucleolar spindle-associated protein 1 (NUSAP1), and CD163 molecule (CD163) were calculated from the 64 specific genes through cytoHubba plug-in of Cytoscape software.

HP levels correlate with an inflammatory state of cytokines and cell-mediated inflammation [24]. As a member of the lipocalin family of transport proteins, LCN2 is well-known for promoting inflammatory responses and as a strong marker for reactive astrocytes [25–28]. DLGAP5 is a mitotic spindle protein that appears to be a target for cell cycle controllers and Aurora kinase A [29]. It stimulates the formation of tubulin polymers and results in tubulin fragments being formed at microtubule ends [29]. CEP55, HMMR, CDKN3, and CDC25C are essential for cell cycle progression [30–33]. PRTN3 is a serine protease found on neutrophils and monocytes and binds to the surface of the endothelium but can also be internalized which induces apoptosis [34]. SKA3 levels affect anaphase transition, which is necessary to divide cells [35]. As an inflammatory mediator, MPO contributes to neutrophil activation, which increases MPO and inflammation in the body [36]. LTF is a protein that is an important contributor to the nonspecific immune system because it helps to limit microbial invasion and maintain iron homeostasis [37]. A spinal nerve ligation model was used to demonstrate that MMP9 levels were upregulated after nerve injury, and then returned to normal within 14 days [38]. NEIL3 has been found to play an important role in maintaining cerebral stem and progenitor cells by preventing oxidative stress-induced DNA damage [39]. NUSAP1 regulates mitosis and chromosome segregation [40]. CD163 is a scavenger receptor specific to monocytes and macrophages and was associated with the polarization of macrophages into the M2 subtype [41, 42].

GO biological processes of the hub genes reveal an intense inflammation response, including acute-phase response, response to reactive oxygen species, acute inflammatory response, regulation of mitotic nuclear division, regulation of nuclear division, hydrogen peroxide catabolic process, and cellular response to reactive oxygen species. KEGG pathways of the hub genes were mainly clustered in the IL-17 signaling pathway. The results of GO biological processes and KEGG pathways of hub genes were in line with many previous studies. Inflammation, oxidative stress, IL-17 signaling pathway, and so on were regarded as indispensable parts of specific mechanisms in SCI [43–45]. There are 93 molecules involved in IL-17 family signaling events in the IL-17 signaling pathway resource [46]. IL-17 signaling pathway contributes to immune response and inflammation in the body [47].

As a small class of short, single-chain, and noncoding RNA molecules, microRNA (miRNA) could posttranscriptionally regulate target genes and is involved in a variety of physiological and pathological disorders, including the development of SCI [45, 48, 49]. The top 10 predicted miRNAs-targeted hub genes were identified as specific miRNAs for a better understanding of SCI, as well as the treatment development.

A total of 10 drug candidates were identified by the hub genes, including dioxocerium, abacavir, potassium persulfate, cryptolepine, lucanthone, oxozinc, 6401-97-4, butein, pimaric acid, and 4,4′-methylenebis. Among the top 10 drug candidates, abacavir, lucanthone, dioxocerium, potassium persulfate, oxozinc, 6401-97-4, and 4,4′-methylenebis could not serve as a candidate. Cryptolepine [50], butein [51, 52], and pimaric acid [53] were considered as potential drug for SCI due to the function of anti-inflammation and antioxidative stress in previous research. Further studies are required to elucidate the impacts of these candidate drugs on the therapeutic effects of SCI.

There are some limitations to this study. Due to the unavoidable patient-to-patient variation, the expression profiles of human blood samples in GSE151371 may have a certain error. Since only bioinformatic analysis was performed, the experimental verification of hub genes and pathways requires further study.

## 5. Conclusion

In this study, RNA-seq datasets related to the spinal cord tissues and blood samples after acute SCI were used for bioinformatic analysis to identify specific mechanisms as compared with trauma without CNS injury. The functional enrichment analysis of hub genes showed a specific immune response. Several drug candidates of hub genes were identified. In addition, the hub genes and the top 10 predicted miRNA-targeted hub genes may be potential biomarkers and need further studies.

## Figures and Tables

**Table 1:** The most significant gene sets between SCI and TC groups in gene set enrichment analysis (GSEA).

Gene set from link to MSigDB	NES	*P* value
KEGG_PARKINSONS_DISEASE	2.22	1.11E-07
KEGG_OXIDATIVE_PHOSPHORYLATION	2.11	9.44E-07
KEGG_PATHOGENIC_ESCHERICHIA_COLI_INFECTION	1.84	1.52E-03
KEGG_GAP_JUNCTION	1.79	5.86E-04
KEGG_ALZHEIMERS_DISEASE	1.75	2.19E-04
KEGG_HUNTINGTONS_DISEASE	1.68	6.23E-04
KEGG_CELL_CYCLE	1.58	2.24E-03
KEGG_T_CELL_RECEPTOR_SIGNALING_PATHWAY	-1.90	2.05E-05
KEGG_PRIMARY_IMMUNODEFICIENCY	-1.99	2.19E-04

**Table 2:** The top 15 hub genes identified from protein-protein interaction (PPI) network analysis.

Gene ID	Description
HP	Haptoglobin
LCN2	Lipocalin 2
DLGAP5	DLG-associated protein 5
CEP55	Centrosomal protein 55
HMMR	Hyaluronan-mediated motility receptor
CDKN3	Cyclin-dependent kinase inhibitor 3
PRTN3	Proteinase 3
SKA3	Spindle and kinetochore-associated complex subunit 3
MPO	Myeloperoxidase
LTF	Lactotransferrin
CDC25C	Cell division cycle 25C
MMP9	Matrix metallopeptidase 9
NEIL3	Nei-like DNA glycosylase 3
NUSAP1	Nucleolar spindle-associated protein 1
CD163	CD163 molecule

**Table 3:** The predicted miRNAs-targeted hub genes.

Term	*P* value	Combined score	Genes
hsa-miR-4750	0.21	7.16	LCN2
hsa-miR-3146	0.13	5.64	SKA3; DLGAP5; CDKN3
hsa-miR-671-3p	0.24	5.60	SKA3
hsa-miR-517a	0.24	5.47	CEP55
hsa-miR-517c	0.24	5.47	CEP55
hsa-miR-4757-3p	0.26	4.76	NUSAP1
hsa-miR-621	0.21	4.06	NUSAP1; MPO
hsa-miR-887	0.28	4.04	NUSAP1
hsa-miR-4259	0.18	3.94	SKA3; CDC25C; CEP55
hsa-miR-95	0.29	3.83	PRTN3

**Table 4:** Drug candidates of the hub genes.

Drug name	*P* value	Combined score	Genes
Dioxocerium CTD 00001451	3.45E-05	3157.45	MPO; MMP9
Abacavir CTD 00003464	7.98E-05	1811.87	MPO; MMP9
Potassium persulfate CTD 00000451	1.20E-04	1386.20	LCN2; LTF
Cryptolepine CTD 00001119	1.44E-04	1235.33	CDC25C; CDKN3
Lucanthone CTD 00006227	6.28E-09	1202.96	NUSAP1; HMMR; CDC25C; DLGAP5; CEP55; CDKN3
Oxozinc CTD 00007012	3.08E-04	752.09	MPO; MMP9
6401-97-4 CTD 00000925	8.22E-03	685.01	MMP9
Butein CTD 00001872	8.22E-03	685.01	MMP9
Pimaric acid CTD 00000440	8.22E-03	685.01	MMP9
4,4′-methylenebis (2-chloroaniline) CTD 00006314	8.22E-03	685.01	MPO

## Data Availability

Publicly available datasets were analyzed in this study. The data can be found here: https://www.ncbi.nlm.nih.gov/geo/query/acc.cgi?acc=GSE151371;https://www.ncbi.nlm.nih.gov/geo/query/acc.cgi?acc=GSE45376.

## Supplementary Materials

Supplementary Materials Supplementary File 1: the differentially expressed genes among groups of GSE151371 and GSE45376.
