# Supplementary material for: Specific Blood RNA Profiles in Individuals with Acute Spinal Cord Injury as Compared with Trauma Controls
Source: Oxid Med Cell Longev. 2023 Jan 12;2023:1485135. doi: 10.1155/2023/1485135 (PMC9851797; doi:10.1155/2023/1485135)
Supplement: Supplementary Materials — Supplementary File 1: the differentially expressed genes among groups of GSE151371 and GSE45376. [file 1485135.f1.pdf]

GSE151371 SCI vs. TC group

Up-regulated gene	Down-regulated gene
ATP9A	TRERF1
FCER1G	PMEL
P2RY1	GPRASP1
ARG1	CIB3
EDNRB	TRABD2A
STOM	TCF7
LTBP1	RASA4
PDGFA	ESRP2
GUCY1B3	LOC102723927
DAB2	LRRN3
RNASE2	MIR4697HG
ABLIM3	BTN3A1
NLRC4	C22orf23
MS4A4A	CD83
UPP1	HSH2D
GYG1	OR52K2
FCGR1A	GOLGA8A
S100A12	LOC101929526
ANXA3	FOXN3-AS2
SDC4	LINC01359
SNX3	MROH5
TFPI	BCL11B
IL18RAP	ITPK1-AS1
MED12L	SERPIND1
MCEMP1	SNORA70B
ZDHHC20	DPEP3
KCNH7	CHGA
CLEC4D	UBASH3A
GADD45A	MUC20
SLC8A3	RPGRIP1
PTGR1	EPHB1
TMEM40	ANGPTL7
METTL9	LEF1
UGCG	SSBP3-AS1
ANXA1	PDE9A
NEDD4	LINC00176
MMRN1	AGAP7P
TLR5	CD28
IDI1	SIRPG
PDLIM1	IKZF3
LINC00989	KY
KLHL2	NRCAM
ASPH	MYH11
RAB32	TSPYL2
MEIS1	LOC100507387
FKBP1B	SYT2
TREML1	JPH3
P2RY12	PLCG1
CCDC150	CATSPER2
CYP1B1	CILP
SELP	SBK1

ABCA13	EVL
CLU	SOX12
CENPE	NKX3-1
MFAP3L	TMEM8B
EGF	SRRM2
WASF3	C1orf95
ASAP2	OBSCN
MIR3916	MIR326
PDE5A	DUSP8
SIAE	SPRN
ARHGAP6	OR52K1
NLGN1	AHNAK2
PLA2G4A	CA6
CYSTM1	TSPEAR
CTDSPL	PRKAG3
CTTN	MIR4313
SAMSN1	NLRP1
TDRP	CXCL8
ELOVL3	MIR641
HPGD	LOC100288152
MIR765	FAM102A
RAB27B	TMEM108
ZFPM2	MFSD6L
ARHGAP18	GRM4
PDE3A	KNG1
LDHA	DTX3
DYTN	LOC104054148
THBS1	MIR342
LAMTOR5	EDAR
EAF2	MIR5685
DEPDC1B	FAM95C
RHOBTB1	FAM153A
SGMS2	ABCG1
PROS1	PLEKHG4
TAS2R40	CPNE9
ARMCX6	SMTNL1
SPARC	TNFRSF25
NAIP	P3H3
MYLK	LOC100129534
PLOD2	ZNF512B
PKHD1L1	CDHR5
PARD3	NTN1
COL24A1	TMIE
ENKUR	ALS2CL
TRIQK	SRRM5
ESAM	CACNB4
SERPINB2	OAS3
TNFSF4	NEAT1
FAM26E	LINC01336
PLCH1	GNRH2
TRIM40	HCG8
GNG11	PDCL3P4
PGRMC1	NPIPB3

COL4A1	MIR647
CASC5	DNAH6
TOP2A	KIAA1683
RNASE1	LOC400685
GNAZ	ACAN
DLGAP5	CDC25B
CABP5	SOX10
DENND2C	MYBPH
PCSK6	LZTS3
BARD1	SCARF1
VEGFC	KIAA0895
SLFN14	RNF212
LURAP1L	CCDC183-AS1
TSC22D1	CYP3A4
BPI	CSNK1G2-AS1
SH3BGRL2	SPDYE7P
ANO2	GTF2IRD1
DTL	NHS
SERPINB10	GRIN2C
FGGY	SCRT2
TUBB1	GDAP1L1
FRMD4B	RGS20
DNM3	PPL
SCARNA22	LMTK3
TP53I3	DFNB59
IGF2BP3	IL17REL
TAL1	PTGS2
FSTL1	NELL2
NAALADL2	WFDC12
CYP1B1-AS1	LINC00565
CXCL5	ADGRV1
GUCY1A3	CA11
LIPN	DLL1
HGF	PABPN1L
PROSER2	MIR3978
CHRFAM7A	TLE2
ANKRD9	NOV
F13A1	MXRA8
C1orf198	MUC5B
C15orf26	MALAT1
PPARG	IFIT3
PLCB4	FCGBP
MMD	PIK3CD-AS2
MIR3945	SCNN1D
LIPH	MIR29B2
NCAPG	PRR15L
SKA3	TSPY26P
C11orf74	TNFAIP2
ACOX2	OR7E14P
CRISP3	LSMEM2
KIF15	LINC01187
SPX	BCL9L
SDHAF3	MYO16

PRKAR2B	STRC
KL	NACAD
TMEM45A	IFNL1
CDKN2C	MIR320E
PF4	THRA1/BTR
PPBP	SCARF2
MGLL	FOXP3
LRP12	TSPEAR-AS1
JAM3	SPEG
MYEOV	MTUS2
CXCR2P1	IFIT1
ELOVL7	LOC728084
TTLL7	SIGLEC1
ST3GAL4-AS1	SLC25A47
LOC101927854	GRAMD1C
VSIG4	MUC6
TSPAN9	CEND1
LINC00884	HSPG2
VRK1	CARNS1
RYR2	ACE
BEND2	LRP1B
KCNE1	A2M-AS1
TRAPPC3L	ANKUB1
PBX1	TRIM60
PRKG1	A2MP1
MYCT1	NAV3
CDKN1A	FGF9
DLC1	SAMD10
LY6G6D	TGM3
IGFBP7	DDI1
CALD1	JUP
KIF4A	MMP28
PDE6H	MIR4718
ITGB3	SORBS3
BUB1	CCAT2
PPAP2B	DFNB31
FSTL4	KRT72
SDPR	A2M
SNORA67	EGR3
LOC105274304	PZP
RGS18	SMPD3
TUBA8	CDC42BPG
SPR	IFI44L
TSPAN33	MYO16-AS1
LOC102724297	IL2RB
EGLN3	GGT5
SNORA71C	PPP4R4
GGH	PRR12
PSD3	PTGDR2
SLC1A3	LOC728752
ACCSL	CHI3L1
KALRN	C15orf39
CA4	PKD1

CCNB1
ITGB5
XRCC6BP1
C18orf65
GRB10
PDGFRA
TRHDE
PDGFC
WHAMMP1
ECRP
AQP10
SLCO4A1
HRAT92
LY6G6F
VEPH1
XIRP2
KIF18A
TPX2
C2orf88
FAXDC2
NCKAP1
RGS6
LCN2
ANLN
SNORD88A
LOC285847
TM4SF1
ADAMTS3
ACSBG1
NFIB
PF4V1
RNU1-13P
RNU4-1
PFKFB2
LOC101927636
PRTFDC1
PTPRF
RETN
RNU12
POLE2
NCR1
SCGB1C1
BIK
CCDC126
SNORD49A
LILRA5
GGTA1P
MERTK
SSX2IP
SYCP2
MMP1
MIR1304
NT5M

CD177
IFI27L1
TPM1
KIAA1462
LAPTM4B
VTRNA1-1
CXCL3
SNORA53
CYP19A1
IGFBP2
GPR84
TPTEP1
ECHDC3
SMOX
NGFRAP1
NUSAP1
WASF1
CMTM5
SNORD15B
ANKRD22
SNORA71A
ABCC4
LOC100507195
SYN2
SNORA7B
MTHFD2L
FAM228B
B4GALT6
RANBP17
CDC14B
ATL1
GPR88
C5orf30
ASPM
DACH1
NXT2
CDC25C
PARPBP
MIR4651
SCARNA1
LOC101929464
TIPIN
ENTPD7
CAPN11
WBP5
SNORA74A
ZMYND12
HGD
CCDC3
EXO1
EPDR1
NAT8B
MMP8

EXOSC4
DPY19L1P1
MAPK10
SNORD12C
SNORD22
ST6GALNAC3
MORC1
MYL9
SNORA45A
SNORA49
ANG
PLBD1-AS1
CLEC6A
ADRA2A
LOC101928306
TDRD9
LINC01262
TRHDE-AS1
FMN1
RNVU1-1
SCARNA16
MIR6780B
DDIT4
SNORA71B
HP
FLJ45079
RNU11
ARHGAP29
AREG
FHL1
PTCRA
TRPM2-AS
HEMGN
SNORA76A
TNNC2
MIR3605
MIPOL1
SPOCD1
TMIGD3
SNORD105B
CPNE5
TMEM255B
DEPDC4
HIST1H2BB
NEXN
GRB14
SCARNA8
TTC8
SHOX2
ENDOD1
RNASE3
BUB1B
GMPR

LOC101928794
HACD1
ADCY6
VIL1
CLEC4G
SEMA6B
CD226
CATIP-AS1
POLQ
PARVB
LINC01572
SH3PXD2B
INTU
LRRC70
FLT3
COL23A1
CRTC3-AS1
ARMC3
LINC00669
FANCL
SNORD55
BMX
SCARNA27
CST7
SNORA34
SNORA70
NEIL3
MIR4772
SLC22A16
SNORA21
HSPA4L
MAP3K7CL
PPP1R14A
KAZALD1
SNORA71E
CARD17
ANKRD18A
LIPC
ADGB
SNORA79
CALCRL
ZNF215
RNVU1-17
CDKN3
N6AMT2
DYNC1I1
MIR3609
GOLGA2P9
SLCO5A1
SNORA37
GPC5
PTK2
IL1R2

SNORA2A
TMEM158
LOC101928075
VN1R1
TGFB1I1
TCN1
PRUNE2
SNORA58
CDKL4
MIR4521
C15orf54
CNN1
ITGA9-AS1
SNORD91B
SNORA22
SNORA38
LOC113230
CLCN1
PCSK9
FLJ21408
SNORA12
TOM1L1
SNORA9
CLEC1B
C1QB
CAPG
RNU4ATAC
LIPM
TNS1
KCNT2
LOC100288846
CEP55
SNORA80A
LY6G6E
LINC00853
CDIPT-AS1
LOC728673
HIST1H2AI
TRPC2
HIST2H2AB
DIAPH3
PVALB
CDCA2
SNORD3C
SNORA40
ME1
PLSCR4
LOC388813
FCGR1C
LOC101927153
SNORA18
DYNC2LI1
SYTL4

SMAD6
CTSE
PHACTR3
NFASC
SNORD68
KCNK13
HMMR
LOC283194
ZNF385D
MIR6837
LINC00534
EPB42
MIR29A
SOX6
BCL2L1
PPP4R1-AS1
LOC102723885
SNORD95
SNORA5A
LGALS8-AS1
ANK2
SNORA23
CD163
SNORA44
ATP2C2
LOC101928063
BAMBI
FBN1
SCN9A
IL18R1
CCDC175
VWA8-AS1
HOMER2
XK
LINC01600
WNT11
RNU4-2
CEACAM6
ABCC3
AHSP
MFSD2B
SNORA16A
CA2
HIST1H2AB
LINC01036
WFDC21P
RSPH9
SNORA73B
HRASLS
SNORA24
KLF1
LOC100506747
LINC00639

TCTEX1D1
SNORA61
VNN1
ITPR1-AS1
LINC00960
SMAD1
SMIM10
LYPLAL1-AS1
EFCAB13
LHFP
ITGA2B
KIAA1211
CDCA5
FAM89A
CASC15
FAM212B-AS1
CRISP2
CDR2L
DOC2B
CDK1
SNORA26
ARMC12
GRK4
AQP1
SNORA10
SNORA6
SPC24
SNORA27
MSC-AS1
OLR1
SNORA64
STEAP3-AS1
CD163L1
SNORD88C
SNORA14B
SNORA65
CENPI
LCA5
PFKFB3
RHAG
ZCWPW2
TMCC2
SNORA80E
DYRK3
CD9
SNORA46
HAVCR1P1
MAOB
SNORA14A
CLEC11A
ENTHD1
SNORD15A
SNORA68

CLEC2L
LANCL3
GATA1
LRRN1
SNORA60
SNORA80B
SNORA20
DNAJC6
TICRR
AKR1C1
FAM171B
ITGA7
SNORA74B
SNORA55
DHRS9
TMTC1
DES
DDX11L9
OR52W1
SCARNA18
PTPRN
GLYATL2
CA1
C1QC
ANK1
KLC3
SNORD13
PTGFR
PTPRD
PRKCDBP
SNORA15
CCDC151
HIST1H2AM
SLC35D3
SNORD66
SNORA66
LINC00309
LOC389831
LAMP5
SNORD8
SNORA62
PLA2G4C
SNORA5C
DDX11L10
MARC2
STPG2
DEFA4
SCARNA11
LOC100289650
SNORD110
SUCNR1
CAMSAP3
TRIM10

SPA17
SPINK8
HIST1H2BJ
CHIT1
ZDHHC19
MPO
SNORA71D
MTRNR2L6
RNU5E-1
SNORD67
STBD1
HIST1H2BM
TARM1
IFI27
SLC39A4
C1orf226
RNU6ATAC
DLGAP1
HIST1H4L
SNCA
LILRP2
CES1
TFF3
LINC00856
LRGUK
ST8SIA6
CCDC176
METTL7B
SNORA8
CCDC34
ID1
HIST1H3B
LINC01151
PDZK1IP1
SNORA2B
SNORD76
H2AFX
LMNA
SNORD99
C1QA
KITLG
GYPB
RNU5F-1
SNORA47
THCAT158
ARHGEF38
CLCA3P
RNVU1-6
GAS2L1
FAM81B
ADAM32
SNORD33
GNG10

SNORA11
SNORD32A
FN3K
BEND6
CLDN5
ARHGEF37
CRYM
SNORA77
FOLR3
RNVU1-7
COPZ2
MALRD1
PCOLCE2
SNORD104
GRHL1
SNHG19
POMC
SCARNA14
SLC26A3
PLEK2
SNORA33
SCARNA4
RNY4
ERC2
TAS2R38
HIST1H2AG
ZNF608
LINC00570
SCARNA3
NECAB1
SNORA52
RNA5S9
HIST1H2AJ
STAC
LTF
SNORD116-18
MIR7641-2
RNVU1-8
MGC39584
SNORD9
PRKAR1B
RD3L
ALOX15B
PRSS57
HIST1H3J
DPPA2
HIST1H3I
SELENBP1
NBEAP1
RNY1
GCOM1
HIST1H2BO
SPTA1

SNORD46
SNORA75
SMTN
GYPA
KCNG2
HIST1H4J
NOTCH3
RNU6-36P
MAOA
LOC344887
SNORD65
TSPAN15
TUBA4B
TUBB2A
LEFTY1
NRGN
SPG20-AS1
SLC9C1
AKR1C2
KCND3
PRTN3
TTK
HIST1H2AH
SNORA38B
SNORA51
REEP1
LRRC32
USP29
LINC00399
SNORA72
SNORA13
RAMP1
TPGS1
SNORA41
SNORA32
HIST1H1B
GADD45G
AP3B2
SNORD45A
HSPA2
SNORD16
OSBP2
DMTN
OLFM4
ALAS2
HIST1H2AE
RNVU1-19
RNY5
HIST1H3F
RNY3
MMP9
RNU5A-1

GSE151371 SCI vs. HC group

Up-regulated gene	Down-regulated gene
QPCT	NFATC2
SLC37A3	ZNF831
S100A8	CCND2
S100A12	GATA3
CLEC4D	PRDM11
ANXA3	TESPA1
IRAK3	ETS1
NLRC4	SLC7A6
KLHL2	BCL11B
LMNB1	DYRK2
SLC2A3	SRSF8
ZNF438	RASGRP1
BST1	TRIB2
IFNGR2	DOK6
LIN7A	ABLIM1
CYP1B1	TGFBR3
PYGL	TNIK
SLC22A4	ANKRD36
TLR5	TRAF5
PGD	LINC00861
FCGR1A	KMT2A
FGD4	RORA-AS1
LILRA5	PRKCH
UPP1	PPM1K
PLBD1	HPCAL4
SULT1B1	KLHL3
PLIN3	SIDT1
NAIP	IKZF3
APMAP	INADL
CYSTM1	NR3C2
ANO10	MAB21L2
ADM	ZBED2
HAUS4	SFMBT1
FLOT1	ELK4
MGAM	LOC102723927
S100A9	SKAP1
TLR8	CBLB
PROK2	MTX3
LTB4R	MTR
ACSL1	KIAA1147
MCTP2	KCTD7
SOCS3	MAF
MCEMP1	STARD9
SLC26A8	ARL4C
CLTCL1	AXIN2
ARG1	AK5
CNIH4	RNF125
TUBA1A	JMY
DHRS7B	SPON1
ASPH	SLFN13
DHRS13	LOC400685

DYNLT1	RNF144A
MARC1	GPRASP1
HN1	MDN1
CSNK1A1L	PLEKHA1
BCL2A1	GNPNAT1
ALPL	BCL11A
BASP1	LINC00342
CD63	AUTS2
FCGR2A	GOLGA8B
GLT1D1	ANKH
ETS2	AAK1
CR1	DCAF16
MAPK14	ZBTB25
NANS	UST
ABCA13	JPH3
MIR223	GVINP1
PLSCR1	CD96
ST3GAL4-AS1	SCML4
CEBPB	DIP2C
GPAT3	MAPRE2
HSPA1B	ZNF320
FCER1G	LRRN3
TXN	CD28
YIPF1	ABI2
FCAR	CCDC141
PFKFB3	ZNF827
F5	TRABD2A
ADAM9	ANTXRLP1
SLC39A1	SLC38A1
SERPINB1	TOX
CKLF	LOC104054148
CSF2RA	HCG18
TMCO3	RIC3
CA4	SSBP3-AS1
SQRDL	CD3G
GYG1	USP24
FCGR1B	TXK
SLPI	UTRN
TBC1D14	CHD6
BLOC1S1	KCNA3
GLRX	RNF43
SRPK1	RORA
ARL11	SLFN5
BMX	PTGDR
KCNH7	FAM120C
HTATIP2	PRKCQ
CARD16	NOG
IMPA2	FNBP4
PGS1	ARHGEF3
HMGB2	MKL2
IL4R	MIR4697HG
CD55	ANKS6
FPR2	YLPM1

METTL9	ZNF37BP
FPR1	SLC1A1
CEBPA	GOLGA2P5
IL10RB	LRBA
SLC25A37	SETBP1
TGFA	PWARSN
MSL3	ZNF26
ACSS2	BACH2
C19orf38	ZNF549
NFE2	FCMR
SLA	ANKRD36B
EXOC6	FOXN3-AS2
RGL4	NFATC3
SDF2	CLEC2D
PLD1	NUCKS1
GCA	LRIG1
SYN2	KLF12
CARD6	CLIC5
ST3GAL4	LOC101927950
MCTP1	TARBP1
NOP10	FAM84B
PLB1	IKZF2
HSPA1A	A2MP1
ROPN1L	ZMYND11
GAS7	RFTN1
DGAT2	DOCK9
IL1R2	ZNF544
MRVI1	NEMP1
MLX	ANKRD36C
CLIC1	TCF7
SMARCD3	CBX5
PPP1R3B	CAMK2D
RAB32	THRA1/BTR
HK3	LEF1
ARHGAP24	A2M-AS1
PADI4	ENOSF1
MMP9	MYBL1
SIPA1L2	FBXO32
WDFY3	OTUB2
TMED8	ST6GAL1
AFF2	CD3E
CKAP4	SEC14L1P1
PPP1R3D	SLFN12L
SLC36A1	NLRC3
EDNRB	DDHD1
MKNK1	ZBTB4
C20orf24	DIEXF
TMEM120A	BCL2
DRAM1	GBP4
KCNJ15	ZKSCAN8
LRG1	PCED1B-AS1
GALNT14	PWAR5
PCYT1A	CNOT6L

S100A11	ZFP36L2
CASP4	PHC1
MANSC1	NTN4
RILPL2	ALMS1
CDKN2D	AQP11
SFXN5	FAM208B
DNTTIP1	ANKRD20A5P
RBM47	SEPT6
HSDL2	HOXB3
STX11	IL24
ALOX5	WWP1
AQP9	MYCBP2
LOC731424	TUB
TP53I3	ITK
TAS2R40	PZP
POR	NCAM1
GRB10	CHMP7
MGAM2	OSBPL3
GNG5	NKTR
PSMB3	PTCH1
MAEA	PASK
GMFG	FRMD5
LIPN	P2RY10
LAMTOR5	MGC70870
RABGEF1	FAM117B
FAM198B	PIK3R1
DDAH2	SLAMF6
MS4A4A	ZNF529
NQO2	PURA
PSMD9	ADAT2
MAP2K6	ZXDB
EMILIN2	HEG1
CRISPLD2	SEZ6L
B4GALT5	SIM2
ACAA1	LOC100507387
S100A6	OXNAD1
CST7	ANGPT2
SIGLEC9	MPRIP
PELO	LINC00987
SLC26A6	CACNB2
VNN2	LINC01128
RNF181	FASLG
RALB	LPIN1
MAD2L2	EDAR
MILR1	RFX7
SRA1	LY9
MIR3605	TSHZ1
ASGR2	ZNF682
CAMKK2	TBC1D4
SLC22A15	EXOG
ALOX5AP	C1QTNF3
CAMP	SMAD3
CEBPD	SNED1

GBA	FAM122B
NCF4	AHNAK2
HRH2	IL2RB
LILRA6	A2M
PNPLA1	USP28
OSBPL1A	HLCS
KCNE1	TTC3
SORT1	TRERF1
KIF1B	CAMK4
PGM1	KLRD1
STK3	CCDC64
NLRP12	GSDMB
MTX1	C5orf63
LOC101928674	IL7R
IER3	NRCAM
STK16	PYHIN1
CAPG	PLCE1
VSIG4	GDF11
CASP5	COLQ
CDKL5	PABPC1
IL18RAP	ZNF594
COL23A1	EIF4B
GNA15	RASGRF2
FLVCR2	ZNF519
SAMSN1	IPW
THOC5	CDK6
TSEN34	CLDN20
PLAU	ZNF736
SEMA6B	ADAM28
KIAA0825	MGAT4A
GPR141	PIGL
NSUN7	PARP15
NEU1	PAIP2B
DNASE1L1	FAM171A1
ITGAM	AKAP5
ZNHIT1	RHOH
ZNF787	COL4A4
IL1RN	IRS1
PPARG	CD47
ST6GALNAC3	SIRPG
LAT2	RNF165
VNN1	TET1
BRI3	LPAR5
ADGRG3	CD40LG
CPNE2	AKAP11
PPM1M	GRHL2
HOMER3	LOC728743
TRPM2	LOC101928100
ECHDC3	SLC30A4
FRAT1	LOC374443
PFKFB4	FAT4
SLC9A7P1	MYO1D
GBGT1	MFSD6

TNFAIP6	DDHD2
RNASE2	EEF1A1
CES1	LAX1
TRIM25	SARM1
MSRB1	STAT4
CACNA1E	MIR600HG
KBTBD7	HOXB4
OSCAR	BCOR
QSOX1	RTTN
TXNDC17	ZNF275
WIPI1	NELL2
FFAR2	MFHAS1
LINC01503	FAM153A
B9D2	SH2D1B
KREMEN1	GOLGA7B
PLBD1-AS1	ZNF251
NDUFB3	CLYBL
JDP2	FAM98B
LY96	ACVR2B
MAFG	PHLDB2
NME8	MBNL1-AS1
FLJ21408	TULP4
PINK1	LRRC37A4P
RASL11A	PROX2
ANKRD22	HAVCR1
OR2B11	DTHD1
SLC11A1	GOLGA8A
FRAT2	AGAP1
LOC102724323	BIRC3
MKNK1-AS1	NCR3LG1
LOC101927851	PTPN4
MXD3	PLAC4
CDYL2	FMNL3
COLGALT1	LOC101929241
LINC00671	AFF3
ZAK	PCED1B
S100P	COL10A1
TLR2	CEP78
TBCB	CKMT2
SEPHS2	ZC4H2
TESC	ZCCHC18
IL1B	EPHX2
RAB24	ZFP62
CCDC71L	PCNXL2
ATP6V1C1	NKAPP1
LOC101927153	TCTN3
PSENEN	CPSF6
SCPEP1	ZNF600
RETN	SH2D1A
IDI1	FAM66C
LPP-AS2	FYN
RIPK3	ZNF507
LIMK2	LANCL1

UBE2J1	HCG8
RRAGD	LOC101928140
DYSF	FAM102A
OSM	SLC41A1
CD177	SLC4A10
AGFG1	ABCB1
SPATA41	SET
SERPINB2	SSBP3
SLC25A24	PRKCQ-AS1
LINC00963	CNKSR2
DRC1	ECHDC2
ACVR1B	LOC101928943
TBC1D2	PRR15L
TUBA4A	CYP4F29P
CMTM2	LOC100499484
FOLR3	EEF2K
CYP1B1-AS1	RRP1B
FUT7	CYP3A5
NFIL3	PHF10
TSPO	ZNF514
LGALS8-AS1	DCANP1
CECR6	MAGEE1
OR52W1	ATM
TNFSF13B	KIF21A
UBAP1	MGA
ATP9A	DOCK10
OPRL1	NCALD
FADD	SLAMF1
SERTAD3	LINC00612
GPR84	TSL
PHTF1	IARS
SH3PXD2B	ZBTB10
RAB20	ZNF204P
MB21D1	MBOAT4
XRCC4	TPD52
CHMP2A	HNRNPH1
PIM3	KLRG1
RNASE1	PPP1R13B
ZNF608	PRKCA-AS1
TMEM255B	HLF
ITPKC	PCDHGB7
LOC101929231	STRC
GADD45B	PKIA-AS1
POC1A	CCDC50
C1RL	ZNF337
LIN37	ELOVL4
PECR	RUNX3
INSC	ZBTB20
CD163	ZNF568
HP	ZNF664
ANG	C1orf21
CD82	DNAH6
RILPL1	SH3PXD2A

CNGA4	ZNF354C
MYD88	PFN1P2
LAMTOR1	C1orf145
IFITM1	YY2
HCK	RCAN3
NFKBIL1	TPT1
PRMT5	HLA-DOA
CD59	MCOLN2
NARF	CYP4F35P
FAM160A2	GALNT12
CHPT1	HNRNPR
ARL8A	GPR174
CLEC4E	LOC100506985
LOC285847	TIGIT
MAPK3	UTP20
PRELID1	FKTN
HK2	LRPPRC
AIM2	STRBP
TBC1D8	MAML2
SLCO4A1	SYNJ2BP
MBOAT2	ZNHIT6
BPI	BTN3A1
SLCO4C1	AMOT
TRIQK	NR1D2
CNTNAP3	LINC01215
FGR	ZNF781
FLOT2	ZNF84
CLEC5A	SLC4A4
MIR8085	DGKK
UGCG	PIK3R3
DOK4	LINC01550
EXT1	BACE2
HPD	TTC12
ECRP	ANAPC1
EXOSC4	LOC653160
PGAM1	TPP2
SIRPD	HNRNPDL
RFX2	LOC101929526
LINC01094	XPO4
TMEM260	SYNCRIP
TMEM91	NCL
ROM1	RRN3
C1orf122	FCRL3
GGT1	ZNF287
HDAC4	TRIM44
RNF10	RASA4
SCARNA22	BTBD18
SNORA80B	TC2N
COX8A	LINC00540
DOK1	PCGF5
PLSCR2	MLLT3
FGGY	LOC100128176
SCARNA20	DPP4

BCL6	PRR5L
DACH1	CXCL8
LOXHD1	LIX1
FCGR1C	OPTN
LINC00482	UBASH3A
SH3BP5L	EGOT
NOL3	OVGP1
NRADDP	FGF9
B3GNT5	ITGA6
PRDX5	FBXL16
LGALS1	TCERG1
PRDM5	LAMA2
OAT	FILIP1L
MYO7B	OTUD7B
KIF3C	DTX3
ANKRD2	KCNA6
JAG1	ZFP3
MYL6	DLG3
ARF5	ADAM1A
SCN9A	ATP10A
BATF	COL13A1
GBAP1	STON2
PFKFB2	EIF5B
AGPAT2	LOC285740
PRKCDBP	ZDBF2
MYBPC3	BTBD11
GPR27	SLC5A3
ASGR1	MEX3C
CD58	LARS
FSTL3	MDC1
TIMP1	CCDC89
F12	GCSAM
MAGIX	ZNF618
CSGALNACT2	B3GAT1
CDA	ZNF235
MGST1	ZNF395
HTRA1	C12orf66
IFITM3	CFAP44
SGMS2	ZNF461
BLVRB	IL11RA
BIK	LINC01389
ZNF230	COLGALT2
CMTM1	ELP2
LOC100128770	DCP1B
TPRG1-AS1	PRPF40B
MIIP	GRAMD3
DOK3	ZNF750
SEMA4A	CNN3
LINC00937	POLR1E
APOA2	RPGRIP1L
PRKCD	EOMES
TYROBP	ALKBH3-AS1
PYCARD	NAP1L3

PPP2R3B	CAND2
SERPINB10	LBH
SNORD12C	CEMIP
RABAC1	CD2
DHRS12	LOC100996286
ATP2C2	RORC
LRRN1	NUDT10
CLRN1	RAG1
CSTA	GFI1
PGLYRP1	TTC3P1
CXCR1	SLC46A1
UBTD1	HIVEP2
ANAPC15	HERC6
NLRP3	TMEM220
ABHD16A	HRASLS5
MMP14	ZFYVE28
FAM214B	THEMIS
SERTAD1	SYTL2
GPR160	KLKB1
RBP7	EVL
TUBB4B	NEO1
PSRC1	DDI1
NFASC	GUSBP4
ETHE1	RAB30
MIR4736	LOC100132111
IDH1	ZNF860
METTL7B	LOC100507424
CCDC159	ALK
CD14	BNC2
COX5B	IPO5
C1RL-AS1	CA6
HGF	APOL6
KIF24	PLEKHB1
ATP6V0D1	FGFBP2
AGTRAP	LOC102723809
UQCRC1	NEMP2
CTSB	MYO6
TALDO1	LINC01336
CHIT1	COQ10A
ARHGEF40	PPP3CC
CCR2	FAM86DP
FKBP5	RPS14P3
C5orf67	TDRKH
MIR646HG	CFAP97
DPY19L3	HEATR1
GADD45A	LOC100506178
HIST2H2AB	TRG-AS1
IFITM2	PDCL3P4
CD300C	RASA3
LILRB5	LOC100507091
LINC00968	ZNF732
MRPL28	ITGA4
LOC645513	CHRM3-AS2

TREML2	MYOT
GSTZ1	LOC100507346
HYAL2	INPP4B
ADCY4	CYP2U1
DIRC2	IQCH-AS1
C14orf2	USP44
PLOD1	BTN3A3
TTN-AS1	FAM153B
SIL1	HNRNPA1
TMEM38A	PRKX-AS1
RAB34	PRKACB
PRSS36	SLC25A4
GAPDH	TGFB3
ZNF254	ADAM12
RDH5	PTPRK
LOC101928163	ANK3
OPLAH	TAS2R43
USP29	TMEM263
OMG	SLC4A7
LOC388282	GPR18
LDHA	LOC102031319
MIR197	NAP1L1
SCN1B	PRR29
FFAR3	ARL13A
TNIP2	HSF5
TCN2	CMPK1
SLC15A3	MSH2
CRISP3	S1PR1
FES	CX3CR1
ATP6V0B	ANKEF1
TOMM40L	PLCXD2-AS1
LSM10	CAMK2N1
PLP2	CD247
DUSP13	IFNG-AS1
KIAA0895L	OSBPL10
C9orf84	ZNF540
ALDH2	ZNF233
CCIN	THBS4
ASNA1	TBX21
ITGA7	SRGAP3
EFNA1	CD83
C1R	SPEF2
PTGR1	NAP1L2
GALNT3	ZNF571-AS1
ROMO1	KCNQ1OT1
GABRR2	LGR6
SPTBN4	OLFM1
GRAMD1A	CXorf57
VASP	AMIGO1
IL18R1	GLS2
ROGDI	TRAF1
IDI2-AS1	LIMA1
FAM129B	NPY6R

KL	SH3YL1
LOC100288778	MDFIC
ST14	BICD1
TDRD9	LINC01278
CATSPER1	CENPV
SULT1A1	PMEPA1
CLEC4A	FAM169A
PQLC1	HOOK1
CHMP3	ZNF439
GJD3	RPL22
MPZ	DLG5
RTN2	SUPT3H
SERPINA1	MID2
MIR6781	AHNAK
LOC102467147	CCDC65
MARCO	ISM1
NEIL3	CCDC136
C11orf42	FTO-IT1
PPP1R27	IL12RB2
PIWIL4	LINC00494
LOC344887	C12orf79
TOLLIP	SPIN4
SNORA5C	MBLAC2
CLEC6A	LDHB
TBKBP1	DDX31
LOC553103	TLR3
KLLN	ARL10
TG	SPATA13-AS1
CDC34	LOC101927827
S100A2	TPH1
GPR108	OR7E14P
CATIP-AS1	RFXAP
LILRB4	MORC4
KCTD11	GNRHR
TMEM144	LOC93622
ECE1	HUS1B
LILRA2	RPS6
ACOT8	PIK3IP1-AS1
MPO	ZNF256
NT5DC2	ZMAT4
COL4A1	RGS20
VAV1	ZNF280B
RASGEF1A	CDC25B
ZMAT5	TTC39C-AS1
FBXO6	LMO7
B3GNTL1	LINC00506
COMMD5	CD160
STX10	FSIP2
LAMTOR2	PA2G4P4
FBN2	KIF2A
WDFY3-AS2	ZSWIM5
TECPR2	CYP7B1
BTNL8	ZNF19

P2RX1	ZFP37
IMPDH1	SACS
LIPM	SMKR1
RSPH14	ZFYVE9
MIR765	IRAK1BP1
MSRA	RFX3-AS1
LRRC4	MYO5B
RNF175	ZNF510
PAQR4	GCNT4
SLC1A3	ALOX12P2
AP3S2	ZZZ3
IFI30	DFNB59
CHRFAM7A	GPM6B
MIR4638	EP400
MIR7848	CCND1
ANKRD35	N6AMT1
TPI1P2	TMEM30B
PARTICL	NSG1
ERLIN1	SLC9B2
CYP19A1	LINC00565
MIR4772	TSHZ2
PLAUR	BCL7A
PLIN5	PAX5
WDR13	ANGPTL7
TNFSF13	EPHA4
UNC5A	MTMR8
FAM151B	SAMD4A
OLFML3	TOMM70A
FAM166A	CACNA1C-AS1
SNORA52	WNT10B
OR56B4	ZNF91
CDC25C	HTR6
LINC01547	KLHL32
KRT13	NDFIP2
HECW2	IGIP
MSRB2	LANCL1-AS1
ARID5A	CELSR1
C9orf106	ADGRG5
ARID3A	ACKR4
DNAJC5	ACVR1C
RPS6KA1	ZSCAN23
SLC16A3	CD2AP
HSPA6	C9orf41-AS1
SH2D4B	ACSL6
ITGA1	ZNF239
RNF217	KLRB1
HIST2H2AC	SBK1
USB1	ENPP5
PTGFR	CYSLTR2
LOC101928977	LEF1-AS1
PMM1	FAM186B
HMOX1	C12orf42
ACVRL1	RAB40B

LOC100131635	APOL3
NTNG2	NPM1
GRINA	RPARP-AS1
BRMS1	COL4A3
PLCB3	GPR183
PHC2	METAP1D
ELOVL3	PDZK1
PLA2G4A	KDF1
ARMC12	C17orf100
TMEM45B	PLCG1
GALNT4	ATP8B2
CATIP	TSPYL5
MIR6837	S1PR5
SIRPB1	LOC101927156
PPP4C	ZNF10
RAP1GAP	NIPA1
WDR45	HIVEP3
S100A5	RRAS2
TAS2R38	ZNF607
LOC100507642	RPL5
FAM157B	ZXDA
FAM20C	ZNF891
OLAH	ICOS
ASL	SAMD12
ORM1	MTERF2
ABALON	TSPYL2
CITED4	ZNF879
FMN1	CCDC152
STARD10	USP36
HOOK2	LDOC1L
SDHAF3	NOP58
BAD	SYBU
TRPT1	ABCD2
LOC101928075	CR2
SLC51A	PHEX
C9orf139	LINC00299
DEDD2	ALOX15
TP53I11	KLRF1
HSD3B7	PARP14
LINC00266-1	SPIN3
HTRA3	LINC00426
DPY19L1P1	ZNF30
SPINT2	OXCT1
ZNF467	PDCD4
HLX	ACAN
NR2E1	CHIC1
SLC5A9	AKAP6
SH3BP5	SPDYE3
MAK	ALMS1-IT1
TMEM11	MBNL3
ACE2	PPP2R2B
ICAM5	HERC2P9
LOC101928306	ZDHHC14

SMA5	ZNF146
ABHD12B	DOCK9-AS1
GRIP2	SMCR5
CDK5	FAM189A1
ARHGEF11	ZNF502
LINC01352	PAPPA
B3GALT4	HLA-DPB1
CLVS1	LOC642943
RELL1	CTD-2201I18.1
MIR4651	PHOSPHO2
LRRC71	COBLL1
CD68	ZNF702P
ITGA9	ZFP82
LTF	SALL2
MMP8	ANKUB1
PDE6H	SEPT8
RAB43	COL19A1
SPG20-AS1	THEM5
RHOG	RAB9B
PRICKLE4	AKT3
CEACAM6	UQCRBP1
ZDHHC19	LOC728554
DNAH17	ENTPD3
SNORD49A	SMPD3
SIGLEC5	NPR2
ST20-AS1	RPS20
STAB1	SORCS3
CDK5R1	TTC21B-AS1
SNORA71A	GZMA
TMEM150B	SPDYE6
LINC01093	RPL6
TNNI2	TSPYL6
ACAP1	CD1C
CLTB	TARSL2
FAM157C	FAM69A
ATP8B4	TRPA1
CSRNP1	UGT2B11
UBE2D1	C9orf117
DDIT4	VPS13C
PSTPIP2	LOC101929089
CYP26C1	CCDC88C
NLRX1	PARM1
NANOS3	RASSF6
TWF2	PPP1R16B
TNFRSF12A	PERP
LSMEM1	ERVH48-1
SH2D6	PKIA
LMAN2	LINC00504
ST3GAL2	OAS2
MYO10	ZNF248
HS1BP3	SYCE2
HIST2H2BF	SNX25
AURKAIP1	MS4A1

CTDP1	RPS3A
SNORA70	LOC102723439
TCN1	RGMB
FAM109B	LOC200772
CEACAM4	KCNH8
EIF6	NPIPB11
GADD45G	PCCA-AS1
C3AR1	TACR1
CXCL16	PGRMC2
MVP	SLC47A2
SNORA47	TMEM237
NAA38	BEND5
B3GNT8	SMYD5
CEACAM8	RPL4
GUCY2C	LRIT3
AP3B2	LINGO2
PICK1	SAMD3
MIR3945	KATNAL2
LOXL1	HEATR4
MERTK	KIAA2022
IRF2BPL	SGK223
NEDD4	FBXL2
LINC01271	LOC101926943
NUDT22	SIGLEC6
LIMS2	ATG9B
TEPP	SIGLEC8
TMIGD3	TH2LCRR
SLC12A9	DDAH1
GNG10	ACOT2
FGF13	BMPR1A
SLED1	PRH2
MYO7A	RPL9
CTSD	ADGRE4P
KCNJ2-AS1	TMEM8B
MICAL1	PRSS33
CDHR2	MSANTD2
CTSG	RPS27A
SHKBP1	TBC1D19
GALE	SLC35F2
ZFPL1	RARRES1
CYBA	SH3D19
SBNO2	PTGDR2
IER5L	LOC101927539
LOC399715	FAM111B
SNORA76C	MICU3
TKT	ATP6V0E2
PCSK9	SPTLC3
LRPAP1	LOC399815
MLK7-AS1	PPRC1
ALDH3B1	PCDHGA11
TUBBP5	CNR2
SYP	ARSJ
CAPN1	AQP3

E2F2	CAPN14
PRR25	TRAT1
TFEB	FCRL6
ADGB	CTSF
LINC01262	IGFBP3
TRPM2-AS	IL23A
TMEM88	CBR3
MROH6	MTUS2
SLC35G6	SDR42E1
HDAC11-AS1	ZNF571
CCDC13	XCL1
SMPDL3A	TMEM75
HIST2H2BA	OR7E12P
SPATA32	PVRL3
SLC22A14	PTMA
TANGO2	MAL
GALNT2	SPDYE1
LINC00264	STXBP1
GYLTL1B	BFSP1
SPHK1	DIO1
ARPC1B	TMEM123
SNORA71C	ZNF583
TRAPPC5	OSTCP1
VPS9D1	RGS9
DNAJC4	LRRC66
FCN1	BTNL9
EML2	TAS2R10
MAP1LC3B2	DEPDC7
ZBTB7B	FAM95C
CFP	SERPIND1
LOC254896	ZNF382
DNM2	APBA1
ERG	SCML1
MTHFS	TCTN1
MIR4420	C17orf78
SMA4	IDO1
NCF1C	LOC100506136
LOC100133985	ZNF23
ALDH4A1	ZNF727
SPC24	CD8A
EAF2	MAGOH2P
CCDC151	MIR342
MIR6738	PPAT
KCNS2	PCDH1
ELANE	MIPEP
SSH3	ACTA2-AS1
LOC388813	RYR3
MAP3K6	LDLRAD4-AS1
TPST1	TAS2R13
TFE3	SLAMF7
DBNL	ITM2A
TOX2	WNT16
WHAMMP1	ESYT1

RELB	ZNF853
MIR5187	FAM186A
CLEC11A	HOXB2
FAM157A	ECM2
MDP1	HS3ST1
GSN	PM20D1
KAZALD1	ARHGAP5
SEMA4B	USP51
AP5B1	GBP5
CCNJL	RASGRP3
TIMM17B	ASPN
LOC100506747	MAMDC2
SKA3	SPATA6L
CCDC126	CD1E
COMTD1	MLLT10P1
IL1R1	FAM150B
SLC35G3	ZNF391
LINC01268	LOC101929452
LILRB3	CD5
HIST1H2AI	ARMCX2
PADI2	HOPX
FBXL19	RNFT2
ADAP1	ENAM
LOC729737	LINC01184
CLCN1	TAS2R3
SIRT7	ADAM20P1
LOC101929464	BANK1
RIC8A	GLB1L2
SNORA25	DNAH11
MIR6892	C2orf88
BCL3	PSMA8
TUBA4B	EBF1
KCNE5	RNF157-AS1
C9orf89	RCAN3AS
C17orf62	RPL7
SH2B2	CHODL
RFXANK	MMD
UNC93B1	TEF
PSTPIP1	SLC16A10
BCKDK	LARGE-AS1
TMEM132D	PGBD1
C1QB	SLC10A5
LINC00266-3	CCR3
GAS6	LOC101928605
LINC01127	PHKA1
RENBP	KLRC4
LTBR	CDNF
SNHG9	ZNF32-AS2
NDUFA13	LINC00238
IFI35	RPS23
TOM1	ANAPC1P1
MIF-AS1	TDRD15
GGH	PIP5K1P1

FOSL2	CPS1
MTVR2	MIR548AR
NUDT18	KLRAP1
SPI1	MCF2L
ADAMTS2	LOC101930452
FAM89B	CXXC4
PPP4R1-AS1	EEF1B2
ECEL1P2	NUAK1
C18orf65	LINC00161
CDIPT-AS1	RPS4X
GALK1	ADAM20
LINC00884	ZFP2
RGS14	MAP3K7CL
CORO6	SLC35G2
CLEC18B	C6orf201
XRCC6BP1	FAM19A1
MIR3916	NEFL
POLR2J	FAM110C
MCOLN1	KCNJ14
FAM131A	KIAA1324L
COL7A1	ADAMTS1
AZU1	BEX2
SNORA78	CYP3A4
GRID2IP	BLNK
RPS19BP1	NTN1
ATOH8	TNFRSF25
TREML3P	MAP9
RAB40C	OSBPL10-AS1
NR1I3	ZNF599
HPCAL1	SPOCK2
HIST1H2BO	C5orf34
GNB2	DNM3OS
SYNGR2	MIS18A
B4GALT2	PLAT
EPHB3	TAS2R4
MOXD2P	KRT73
MPST	OMD
HIST1H4J	MIR3679
PRTN3	OR7E2P
SLC28A3	BDNF
DRC7	ZNF285
ADCK4	SLC29A2
SAMD15	CD3D
STXBP2	C1orf177
ATG101	MCOLN3
LY6G6D	ABCB4
SLC22A16	PDE9A
SNORA34	RPS27
LAMB3	AGBL3
LOC100289511	LOC102724596
C10orf131	COL5A2
TCEB2	KCNAB1
TYMP	LOC101927557

HIST1H2AB	ENPP3
UPB1	SPATA9
TST	IL5RA
EIF4EBP1	DIRC3
LAMB2	PDGFD
TOR4A	ZNF208
C17orf53	ADAM23
ABHD8	CTAGE7P
LYL1	FAM129C
ZNF670-ZNF695	CTHRC1
MAOA	IGFBP4
LOC730202	PTGS2
DCXR	CARD11
A3GALT2	DBH-AS1
HIST1H2BC	PDZK1P1
OLR1	LMF1-AS1
KIAA1462	ABCE1
HCFC1R1	SLC35F3
GPER1	NT5C1B
CLEC3B	ANGPTL3
GSDMD	AJAP1
BCAT1	CXCL10
DEFA4	ELOVL6
S1PR4	OCIAD2
STEAP3	TRMT13
NOXRED1	ZNF610
CAPNS2	IQCH
CYHR1	KY
CDADC1	DEPTOR
CDH26	LINC00925
FLT3	CPLX2
HIP1	SOX13
LST1	RPL21
CAMKK1	ASS1
CUEDC2	GSPT2
UBE2S	CATSPER2
ZNF429	ZNF501
ENTPD7	ZNF711
IRF7	ABLIM2
CLEC4G	SRRM2
CHRNA2	CCDC38
ZNF775	RLN2
SEPT5-GP1BB	RPL35A
IGFLR1	DPH6
HIST1H3G	TAS2R31
HIST1H2AC	IMMP2L
TTLL12	LOC100996579
CFD	MFAP3L
CIRBP-AS1	HKDC1
ZNF282	FAM86C2P
CERS4	RPS12
SEZ6	ZNF165
SYCP2	THOC7-AS1

GPR37L1	NT5E
CYTH4	FAM86C1
SNORD88A	TMEM133
KIRREL3	KIAA0125
BTBD19	TMEM182
HIST1H4H	GZMK
SPATC1	CCR8
HIST1H4K	NUDT9P1
LRRC70	LDB2
DPM3	PTPRM
MIR1976	CCNI2
DOC2B	LARGE
RILP	ACOT4
UNC45A	TARP
FLJ20021	C10orf35
PPP1CA	RPL30
SNORA80A	ZNF572
HIST1H3C	MTUS1
ABTB1	FRG1CP
CEACAM1	PCAT29
ANLN	E2F5
CNTNAP3B	SH2D2A
MIR6847	KLRC3
MIR4449	CLIP3
SECTM1	HOXB-AS1
JAK3	CACNB4
JUNB	SDPR
RAB3A	GPR68
NCF1	LOC400553
LINGO3	CFL2
LOC102546294	PDE6C
HIST1H1B	ZNF883
LINC00856	CDHR1
ID2-AS1	LOC101928696
SOWAHD	RLN1
NUSAP1	CACNG6
SNORA71D	RDH16
GLMP	SNORD102
LOC101927795	FRMPD3
FERMT3	LINC01108
SCNN1A	CAPN5
TMED1	HNRNPA1P10
MIR1250	KLRC1
IL1RL2	USP18
DHCR7	RPL3
MFSD7	TMEM108
GLTPD2	ZNF790-AS1
MIR4523	OR2A4
TNFRSF8	CCR7
SDK1	SGPP2
SNORA73A	MALAT1
CCM2L	DFNA5
C16orf93	USP30-AS1

ICAM3	LOC650293
ADAMTS3	OLFM2
ZNF593	FABP3
MIR372	KCNIP4
DOCK4	ZNF300
HIST2H3D	XKRX
MIR373	LRRC39
TRPV4	BHLHB9
ENKD1	PRKAR2B
KCNK5	LOC101929125
PPP1R1A	ANKRD20A12P
PDLIM7	MIR155HG
IGFBP2	ADH4
G6PD	FBLN5
LOC101926963	LOC100129216
POLR2L	LOC101927972
MIR7845	LOC100507388
ZNF408	NUDT11
SPR	SLC38A11
UNQ6494	GARNL3
CRACR2B	MSX2P1
JOSD2	FCER1A
GRK6	RHOXF1P1
HIST1H3D	TMEM150C
COPE	C12orf75
C4orf48	LINC00511
EHD1	GRAMD1C
SNORD43	TLE2
FDXR	STAG3L5P
MPV17L2	FAHD2CP
PRG2	FCRLA
BCKDHA	SPRN
MIR1273D	ENHO
CCDC9	LOC285593
GRN	OR52N4
MIR6832	BCORP1
FCGRT	BEND4
METRNL	TIMD4
NECAB1	TRIM2
SNHG19	HLA-DRA
GADD45GIP1	LOC101927391
NAPRT	C2orf71
COMT	KCNC1
HIST1H2AL	GZMB
ASPRV1	BOK
FAH	HEATR9
MIR646	ZNF229
KDM4B	AGAP9
CHMP1A	KCNQ3
MIR4257	SDK2
VAMP5	LOC102724484
AFAP1L1	SLC38A4
LOC100996693	FAHD2B

SNORA67	MYCBPAP
MIR29A	PRICKLE2
OLFM4	SCN3A
SHOX2	SCART1
LOC101929897	LINC00515
HIST1H2BN	RAPGEF5
LOC101928093	FAAH2
LINC01272	OLIG2
VWA8-AS1	SMIM18
ZDHHC12	SYT17
COL9A3	RAI14
SF3B5	KLHL14
SCAND1	CPA3
GPR87	AFAP1-AS1
ACADS	CDH12
PNPLA2	ACMSD
CAPN13	PPIAP30
PLTP	ZNF192P1
ZMYND15	LOC101928767
TADA3	SGCD
ENDOG	LOC101927412
RELT	POU5F1B
HIST1H4I	FAM132B
TTC8	GPR182
PPP1R14A	NBEA
RBM42	BHLHE41
CAND1.11	ARHGEF35
ALOX15B	TMEM14A
C19orf67	SPON2
GK3P	LOC100289561
LOC100289650	SORBS2
INHBB	CUX2
GYS1	SCAMP5
APOBEC3A	PRSS2
DDIAS	CRYGS
MOSPD3	ADGRB2
C18orf32	PRO1804
ZP3	CRB1
FBXL15	LINC00943
PLIN4	LOC100506124
SHARPIN	PDGFRB
FLII	TMEM99
NDUFA11	DNASE1L3
LINC01023	PEG10
HPGD	TMEM177
ADAM8	DDR2
TMTC1	OGN
HDGFRP2	FAM24B
TSSC4	GTSCR1
SESN2	PTPN13
SLCO5A1	LINC00865
KCNG2	FCGBP
LRP3	MIR573

R3HDM4	PODN
PGPEP1L	RBFOX2
DHX34	NOV
RPH3A	LCK
KLF5	SPTSSB
LOC100288846	MIR326
OXER1	FLJ43315
NUCB1	MYZAP
PPEF1	CFH
TSR3	LOC101928279
ZC3H12A	GPR135
PROKR2	OCLM
MIR618	STAP1
SCARNA12	CA2
SCYL1	TPRG1
ARHGEF17	GTF3C2-AS1
LRRC25	ZNF32-AS1
RNU86	LYG2
IKBKG	DLL1
GCAT	NINL
3-Mar	PCDHGA9
PGLS	CBR3-AS1
LINC00659	CFAP69
VPS28	C9orf153
MMP24-AS1	COL5A1
ARHGAP29	CD6
OR52L1	EXTL2
RNASE3	PDIA3P1
INSL3	FGFBP3
MIR6753	GFRA2
SNORA5A	RPGRIP1
AATK	LINC00883
LOC101928530	KLRC2
PARD6A	C8orf46
FLJ45079	MEOX1
SNORD74	LRP1B
FAM110B	SLC16A14
MYLPF	HLA-DQA1
HIST4H4	HRNR
PCOLCE2	TAS2R14
RUSC2	LINC01535
UBXN6	ZNF704
RIN1	MIR1254-1
LINC00607	PCDHGB6
PPAP2B	LOC105372795
MIR6813	CDK15
SDF4	ANKRD18B
MPP3	WNT10A
EFEMP2	LOC644554
ARRDC1	ZNF157
ZNF503-AS2	UGT2B28
HIST1H3F	TRPC1
CD151	ADRA2A

DDX39B	PCDHGB8P
ACOX2	GDAP1L1
RNF151	TRPV3
SNORA64	LOC100130880
KAZN	ADAMTS10
DLGAP5	SOCS2-AS1
MIR6842	RNU6-57P
TFF3	SH3BGRL2
EMX1	GOLGA8N
PRSS8	ANKRD20A8P
MAP2K2	USP12-AS1
ARSE	TNFRSF13C
NDUFA3	LOC101927190
UFSP1	CMSS1
C1QA	AFAP1
LCN2	ARHGAP44
CHCHD5	ZNF705E
RAB19	TAS2R50
ADORA2A	LOC728989
MIR6826	IL7
CCDC153	LMOD2
RASD1	TMEM200A
ERCC5	DACT1
TERC	CLC
EPHB2	CXCR5
LOC100507006	TMEM221
LINC00999	NLGN2
LSP1	IL4
DAPK3	KLHL13
LILRP2	GATM
IDH3G	FZD6
RGMA	RNU6-19P
MIR6075	CCAT2
PIN1	SYN3
FABP6	RPSAP58
POLR2M	PCDHGB5
VPS9D1-AS1	ERI3-IT1
SDF2L1	IFNG
ZBTB17	LOC283788
DEPDC1B	C2orf27A
STAB2	EGR3
RPUSD1	GCOM1
SMG1P5	ANKRD31
TULP2	KGFLP1
LYPD8	C3orf49
SLC19A1	KRT72
ABCD1	LOC101928896
ICAM4	KDELC1
SNORA7B	STOX1
MIR141	NAV3
TCIRG1	CRLF1
2-Mar	ADGRG6
SNAI1	ZNF826P

SELO	SLC25A23
TREML5P	GRIN2C
NTSR1	LINC00920
CLRN1-AS1	ADAMTS5
H3F3AP4	SNORD20
CDKN3	OR7E37P
YIF1B	TXNRD3
HIST1H3J	DIP2A-IT1
H2AFX	SIRT4
SH3RF3-AS1	ARHGAP20
FAM132A	LOC728084
PLEKHJ1	SCN7A
CLEC18A	ST7-AS1
FAM3A	S100B
GPRIN1	THBS2
MIR30C1	MIR548P
DLC1	ACSBG2
TRIM9	GAS2
CYC1	CHN1
TBX6	PTPN20
RAB1B	ARHGEF10
TGFA-IT1	SRRM5
MED16	LZTS3
P4HA2	CHAD
SPATA2L	MST1L
SNORA68	SPARC
MIR191	SIK1
HSPA4L	EFCAB6
HIST1H4B	MDGA1
NCKAP5L	MIR186
RNU1-13P	STEAP1B
TMEM115	ISPD-AS1
CD300LD	PRF1
IL17RC	BCL9L
ETFB	KCNQ5-IT1
HIST1H2AJ	HGD
PRUNE2	TRIM51
HIST1H2BL	ALPK2
GUK1	ACSM3
XKR7	SOGA3
MIR4673	HIGD1B
INAFM1	PCDHGB3
SLC22A18AS	GPR19
KIF4A	CADM1
OSGIN1	ZNF683
GTF2IP20	ZNF311
MIR3124	TGFB2
SLURP1	UBXN10
ESRRA	LOC392196
SNORA57	WNT7A
SMTN	LMO7-AS1
NATD1	PID1
FAM45B	ENKUR

CAMSAP3	LOC100507600
GPR17	DOCK9-AS2
NCR1	ZNF703
PCP2	CLIC6
GSG1L	APOBEC4
OR52B2	TNK1
TFPT	TUBB1
AIP	LOC389834
SPACA6P	LINC00544
KATNB1	SOX8
HIST1H4D	C1QL3
MIR5690	PCDH17
ZNF219	PCDHGA10
OLFML2B	PROX1
TMEM8A	ALDH1A1
LOC101929412	KLRC4-KLRK1
VPS18	HERC5
MIR6819	MYO1B
MRPL23	REREP3
MIR3175	PACERR
OIT3	LOC283194
ADAM30	ESM1
ITGB2	LRRC7
C16orf86	MMP28
ID1	MLF1
SSPO	KLRK1
FKBP8	CA11
MYBPHL	CACNA1I
CHST13	TMEM56-RWDD3
DES	LDHAL6B
EFCAB12	SMTNL1
SLC22A18	PLEKHA7
CCDC64B	PTPRG
FAM195B	LOC100129316
SNORA32	FRMD6
TMEM176A	LOC101927560
KCNQ1	ABCA8
C1orf226	ZRANB2-AS1
LRWD1	METTL21C
POLD1	HDC
SNORD88C	SORBS3
SH3GL1	ETNK2
ADAM15	IL12A
CDS1	PRSS35
KCNK13	MEIS2
SPINK8	LOC101927179
TSSK6	CDH2
FLJ23867	CARNS1
RSPH9	TMPRSS11D
TMEM160	SMARCA1
NDUFB7	RAB27B
LRCH4	HOXA10
MMEL1	SNORD69

LINC01600
DEAF1
HIST1H3B
GTF2H2C_2
IL27
FURIN
CDCA3
SEMA3B
BSG
ANPEP
ANKRD13D
MIR8072
LINC00536
VWA7
TARM1
SLC2A14
MMP25
RASGRP4
PTRH1
MIR140
GPAA1
NOS1AP
PTPN6
GTF2IRD1P1
TPGS1
ADRM1
LINC01581
SNORA73B
CNN2
SNORA10
PEX16
HIST1H4A
NLRP6
EMILIN1
FAM89A
ACOXL
LOC102724958
TMEM132A
LINC00399
GPRC5C
HIST1H2AK
PRSS27
IFITM10
GJB6
APCDD1
SCUBE1
CYGB
DPPA2
GAA
COL4A2
LINC00639
ATG4D
LY6G6C
ZNF334
SNORD19
CADM2
FREM3
PARD3B
SNORD19B
LOC101928834
C12orf40
INTS4P2
MIR4458HG
CORO2B
ROBO1
C15orf54
ATP8A2
LRFN3
CD74
MACROD2
RNY3
ARHGEF28
KITLG
TNFSF11
COL5A3
PMP22
PRKY
PPP4R4
PPBP
GZMH
HRK
OBSCN
CXCL5

CD163L1
C15orf65
ARMC6
LINC00906
DAAM2
EGFL7
HIST1H3I
ZGPAT
C20orf195
TMEM95
HPN
MAP1LC3A
MIR657
NDUFS7
SSNA1
MIR26B
WDR18
HIST1H2AH
PAFAH1B3
NDUFS8
NUBP2
LPPR2
MICALL2
MPG
HIST1H2BB
CARD17
MRPL54
RPPH1
SCARNA2
MBOAT7
RNASEK-C17orf49
HIST1H2AM
MRPL12
KLF1
MVB12A
SNHG25
SMIM10
GOLGA2P9
ABHD11-AS1
HMMR
SAC3D1
MAP3K11
HIST1H2BF
ATP6V0C
PDLIM4
ATP5E
COL18A1
LENG9
GPX1
GPR137
C9orf163
NR1H2
PHPT1

TRABD
GPX4
CARD9
AREG
STBD1
PNPLA6
HAAO
SERPINA2
CORO7
MAPK11
LRRC6
C11orf68
LOC400927-CSNK1E
SNORA16A
FAM173A
UBXN11
NFKBID
SIPA1
ZC3H3
PNKP
STUB1
RARA
SNORD10
MT1JP
PGF
FSCN1
TAS2R39
NCLN
LRFN1
C1QC
RCVRN
LAMTOR4
APOBEC3B
NBEAL2
EIF4EBP3
APBA3
SLC52A2
CCDC157
TNNT1
ECI1
SNORA24
ALDOA
SULT1A2
KEL
LINC01490
GLP2R
UNC13D
CEBPE
POMC
SH3TC1
FSTL4
C9orf152
CHAC1

SLC4A2
NCF1B
SMIM1
RHCE
LINC01337
DGCR6L
RBCK1
SIRT6
C19orf25
SNORD37
SCO2
BLACE
ARHGAP4
NRN1
HIST3H2A
PANX2
CORO1A
LHFPL3-AS2
MIR6126
PGLYRP2
MAP1S
CEP55
NINJ1
TUBB2A
NOSIP
NECAB2
PLEC
HAVCR1P1
DEFA3
EVA1B
SYTL1
MVD
EME2
A1BG
NUDT3
TEN1-CDK3
ZDHHC24
NOTCH3
CSF3R
RD3L
HIST1H3A
HIST1H4L
RNVU1-7
MAFK
CORO1B
SLC38A10
KLF16
DNASE1L2
TLR9
LINC00664
MIR3138
IFI27
SPNS2

SCARNA21
CR1L
HNRNPH2
SLC6A9
SNORA74B
INHBA
SPSB3
RAB26
PGAM2
MARC2
MYBL2
ABHD17A
ODF3B
RAI2
CYYR1
SAMSN1-AS1
ITGB4
LINC01002
MBD3
HIST1H4F
KLC3
ABCA2
SNORA71E
LRFN4
RUVBL2
TCTEX1D1
HPYR1
DOHH
RNVU1-1
TIGD5
GPR35
HIST1H2AG
FBXW5
ZNF524
ABHD16B
RN7SL1
SPNS1
NME3
RASSF1-AS1
MPND
MLST8
TMEM176B
MRPL41
SNORA21
TSC22D4
CLEC12A
DHRS9
FAM195A
LVRN
CCDC107
TELO2
IFITM5
HELZ2

MIR4451
CC2D1A
C4BPA
PITPNM1
HIST1H2BH
HCG4B
SNORA71B
E4F1
TSPAN4
C19orf24
HIST1H2AE
SNORA45A
HSPA2
C9orf16
PRAM1
CDK2AP2
CCDC88B
SNAI3
RNU6ATAC
DPP7
SNORA15
SCARNA16
HIST1H4E
ZNHIT2
CMTM5
RNA5S9
CCDC85B
ZYX
ANKRD34B
KCNH3
HIST1H3H
PARP10
PER1
HIST1H2BI
SIVA1
LY6G6F
RN7SL2
COL9A2
ABCA7

GSE151371 TC vs. HC group

Up-regulated gene	Down-regulated gene
QPCT	STON2
SLC37A3	MFAP3L
GLT1D1	MMD
S100A9	DAB2
SLC22A4	C2orf88
ACSL1	PRKAR2B
S100A8	SDPR
ARL11	LIMS1
ANKRD2	SH3BGRL2
MGAM	SPARC
SMARCD3	ELOVL7
CR1	LINC00989
ALOX5	ASAP2
CRISPLD2	MAPRE2
PYGL	MAP3K7CL
SLC2A3	RAB27B
LINC01503	ENKUR
BRI3	TUBB1
RAB24	CXCR2P1
SLC36A1	PDE5A
TUBA1A	WASF3
ADM	PGRMC1
CLEC4D	TSC22D1
ICAM1	RNF11
PLIN3	LPAR5
NRADDP	MEIS1
MARC1	PDGFA
PLAU	PTGDR
PADI4	GUCY1B3
KCNJ15	NLGN1
IRAK3	GNG11
ALPL	DNM3
BST1	HOXB4
PLAUR	BMP6
FFAR2	MYLK
WDFY3	SH2D1B
SLC11A1	CDC14B
PROK2	TDRP
CAMKK2	PPBP
DGAT2	TRIM40
FABP6	KIF2A
LOC100128770	ADRA2A
NLRP12	ABLIM3
LINC01134	ZNF542P
FCAR	CA2
KREMEN1	DOK6
PGD	PROSER2
FPR1	HGD
SLC26A8	CXCL5
SOCS3	ARHGEF12
HRH2	ARHGAP6

IL1B	SAV1
ANXA3	FHL1
S100A12	DAAM1
PGS1	F2R
SFXN5	MBNL3
LILRA5	GUCY1A3
LINC00963	PBX1
IL4R	C1orf198
GAS7	GGTA1P
C1RL-AS1	ANKRD36
CCIN	CD226
CDA	LOC101927854
MTX1	MYCT1
HAUS4	AR
LOC401052	ENDOD1
GPAT3	MAF
ECE1	ZC4H2
APBB3	LTBP1
HCK	EGF
FLOT1	CYP4F29P
MYL10	ARHGAP21
APMAP	RHOBTB1
F5	P2RY12
PIM3	JAM3
SLPI	GFI1
IL1RN	CTTN
NCF4	SH3TC2
SPATA32	CCDC92
LOC100996351	BEND2
LTB4R	ZNF367
MYBPC3	MOB1B
PFKFB4	ZMYND11
SLED1	TFPI
GBGT1	SLFN14
PNPLA1	SPIN4
TMEM38A	NAP1L1
LIMK2	MSC-AS1
LMNB1	IGF2BP3
C1RL	LOC283194
CECR6	PLA2G12A
CEBPB	CNST
CEBPA	ZMYND12
ACAA1	PF4V1
PLBD1	CALD1
PYGM	PKHD1L1
CLK3	TNFSF4
RILPL1	RNF165
TMEM45B	FAT4
LIN7A	KALRN
GALNT14	RGS6
BLOC1S1	RSU1
MTVR2	MED12L
HK3	ZXDB

NFE2	C15orf54
ZNF516	LRBA
MGAM2	MYBL1
LILRA2	NFATC2
CD14	GGACT
CHMP2A	CTDSPL
S100A11	FASLG
MIR197	PLCB4
HSPA6	PDLIM1
BCL6	FSTL1
SH3BP5L	DTHD1
JDP2	KLRB1
CSRNP1	ZNF831
MMP9	TGFBR3
LOC100506801	VANGL1
KLHL2	GATA3
GBAP1	LRP12
STARD10	F13A1
DNASE1L1	NCAM1
FRAT2	GNAZ
CPNE2	DDAH1
ANPEP	LURAP1L
C19orf38	ZFPM2
FAM157A	DYTN
CLEC18A	SLAMF6
FCGR1B	ZNF727
CADM4	LIPH
AGMO	EGLN3
UBTD1	KIFAP3
FAM157C	MTURN
PLSCR1	SLFN12L
ADCY4	LOC100128176
ETV4	ANO6
SCN1B	PSD3
C7orf61	TMEM64
NCF1C	ODC1
HOMER3	BCL11A
GPR27	SLC1A1
DHRS12	AQP11
ATP6V0D1	THRB
LOC100288778	FHL2
OTUD6A	CMPK1
DYSF	SPX
MSRB1	PDGFRA
CCDC64B	PIK3R3
CKLF	PEAR1
LRRC4	PCGF5
CACNA1E	C1orf21
RFX2	AKR1C8P
SLC35G6	PRKG1
LIN37	PDE3A
ST3GAL4	SVIP
DNTTIP1	AMIGO2

CYP1B1	RYR2
PLB1	PROS1
S100A2	AKR1C3
RSPH14	GCOM1
FCGR1A	ETS1
INSL3	CDK2AP1
SLC15A3	ZNF385D
TMEM132D	AUTS2
SULT1B1	SSX2IP
PFKFB3	COBLL1
CCDC36	DCP1B
GRIP2	NUCKS1
COL7A1	C15orf26
PLIN4	RASGRP1
MAPK14	BANK1
TECPR2	P2RY1
CIB3	MSANTD3
B3GNT9	SLC8A3
NLRC4	LINC00883
ART1	RFX3-AS1
DENND3	PF4
TUBBP5	LAMA2
TSEN34	LINC00299
IFITM2	ITGB3
LOC101927851	PPP1R13B
KIRREL3	WEE1
DHRS13	MYZAP
CHRNA2	VPS13A
ADGRG3	KIAA1211
LTBR	ARL4C
PLA2G4E	LOC101927827
SLC12A9	CCSER1
ITGAD	TLR3
TLR5	ATL1
SEMA4A	BTG3
DSC3	B4GALT6
CAPN13	SPTSSB
RGL2	ZNF823
LINC01317	NCKAP1
BMX	ANKRD36C
IMPDH1	SCFD2
SLIT1	ARMC3
LOC731424	GRAP2
LINC01105	ZNF664
CORO6	RORA-AS1
FGR	TLE1
LRG1	TRAPPC3L
SIPA1L2	CLYBL
VASP	TAL1
TG	RAI14
CEACAM3	RGS18
MIR372	RAB30
USP35	LOC101929340

MIR4257	GRB14
NANOGNB	NAALADL2
GPR37L1	CABP5
LOC553103	EVA1C
HOOK2	PRKACB
STX10	LOC105373300
TONSL-AS1	WHAMMP2
CYSTM1	MAP4K5
NECAB2	PRICKLE2
MIR223	TDRKH
PLAC9	LOC100507195
TMEM120A	PHLDB2
OR2B11	KLRF1
MAPK3	SPAG8
GNMT	KCNA3
TMEM119	ANO2
TALDO1	TRIM58
MANSC1	HEMGN
NAIP	PLEKHA8P1
DNAH17	GSTA4
ICAM4	YY2
OSCAR	CLU
SH2D4B	PTPN4
RNF151	HOPX
GBA	ZNF600
DHX34	CCDC175
CYP26C1	CAMTA1
SSH3	CXXC4
DRC7	ADAMTS6
FAM160A2	CAMK2D
PRKCD	RAG1
AGTRAP	TPT1
PCDH11Y	IRS1
ARHGEF40	MORC1
FAM214B	RGCC
LILRA6	TMEM158
IFITM10	SDC4
CEACAM4	LINC00861
SIGLEC9	SKAP1
GAB2	RGS9
VAV1	ALKBH3-AS1
TFE3	ADAM12
NHSL2	PALLD
PINK1	CCND1
LILRB5	LINC01011
LOXHD1	CEP78
ROPN1L	FAM26E
SLC16A3	KITLG
DRC1	RNY3
MKNK1	ZBED5-AS1
NTNG2	NCALD
NACAD	GOLGA8B
TBC1D2	LOC101928140

ST3GAL4-AS1	SLFN5
CCNJL	SMKR1
TMEM52B	HOXB-AS1
SLC22A1	PLCH1
EXT1	RGS10
SIGLEC11	ANKRD20A5P
FAM157B	LYPLAL1-AS1
CYB5D1	PDE4D
SEMA3B-AS1	CLIC5
CSNK1A1L	DGKK
KIAA0319	C1QTNF3
CNTNAP3	MKL2
CPT1B	NGFRAP1
C9orf163	LOC101927636
FAM132A	ZMAT4
SEMA3B	BET1
ST3GAL2	C1orf116
DNAJC5	PRTFDC1
NOL3	MLH3
GRAMD1A	LOC200772
TMEM88	GRHL2
GRM4	SELP
CA4	VEGFC
SYN2	ZNF732
LILRB3	PRMT6
BTBD19	SLC38A1
FUT7	TOX
CDKN2D	ZNF860
RTN2	C12orf75
KCNQ1	ISCA1
LRWD1	PTGS1
RAP1GAP	PVALB
FITM1	PBDC1
F12	CYP2U1
BCL3	CTHRC1
ATP6V0B	ZCCHC18
EFCAB12	TTLL7
IFITM3	LOC102031319
LINC00632	TMSB4X
OPRL1	HRASLS5
DOK1	MYO1B
POU5F1P3	ST7-AS1
UCKL1-AS1	MMRN1
UNC119	ZBED2
ATP1B4	PARM1
KIAA1683	LOC653160
GYLTL1B	ZNF256
APOBEC3A	LINGO2
NFE4	RORA
SLC22A13	CYP4F35P
PPP1R27	LOC101930452
TSPO	ARHGEF35
YBX2	FAM86C2P

SLC19A1	PTK2
B9D2	ITGB1
ARID3A	PTCH1
NADK	DCLK2
TNFSF13	SOCS2-AS1
PHC2	FAM46C
RELT	PYHIN1
TOM1	CBR3
FBXO6	ST8SIA6
NCF1	CCDC3
ITGAM	ITGA9-AS1
SYNPO2L	PARD3
CXCR1	CHODL
LINC00383	MGLL
DOK3	TARSL2
MIR647	AFAP1L2
ZNF467	ADAMTS1
TGM3	OPTN
LOC100287792	ENTHD1
LOC101928266	ARHGAP10
EHBP1L1	KCNA6
OSM	CEP290
LOC101926911	MLLT3
MVP	SLC4A4
CYTH4	PCDHGB8P
HS1BP3	SH2D1A
MIIP	FILIP1L
MCEMP1	ENTPD5
C5AR1	CAAP1
AGPAT2	LOC101928834
ATG16L2	ZNF354C
COX8A	DFNA5
A3GALT2	ABCB1
C5orf67	GPR88
MIR6124	COL13A1
TRIP6	FAXDC2
C9orf139	MAGEE1
LINC00887	ARHGAP18
BCKDK	ISM1
LOC101929412	ABCB4
TGFA	COL24A1
PLCB3	ST3GAL3
B3GNT8	HPCAL4
PYCARD	PDGFD
ENKD1	HOXB3
ADAMTSL4	FCRL3
SHKBP1	FGF2
GPR108	TET1
LOC102724323	FAM169A
MMP25	ZNF569
FES	KHDRBS2
TP53I11	RASGRP3
SPTBN4	FGFBP2

LOC100289511	CNKSR2
HPD	CCNB1IP1
LOC100996291	TNS1
LINC00092	MS4A1
AP5B1	SPON1
UPP1	XCL1
ASGR2	PKIG
IL27	GPR89A
PTPRG-AS1	BNC2
ZBTB7B	FBXO7
PISD	P2RY10
APOA2	MIPEP
ANKRD13D	TIMP3
EGFLAM-AS4	CDNF
LOC101929897	EBF1
IRF7	FAM81B
DEDD2	PTPRK
CKAP4	LOC100506178
PPP4C	LINC00238
SNHG9	TBC1D19
ARHGEF11	ANGPT1
NINJ1	CCNB3
GGT8P	BIRC3
GDPD2	RHBDD1
SERPINA1	TMEM237
GFRAL	USP12
CD82	TM4SF1
DOCK4	SLC35D3
LOC101929550	LOC101241902
PGAM2	CD96
XKR5	ALG10
VPS9D1	TMEM40
IER3	SOCS2
POC1A	CCDC141
MAP3K6	ITGB5
CORO7	BNIP3L
ALX3	GRHL1
LINC00482	USP44
ARRDC1	IL7R
UNC5A	ACTR3B
LINC01352	UGT2B28
RELB	SEZ6L
PLIN5	PCDHGB7
TAS1R1	PMEPA1
PDLIM7	COL19A1
QSOX1	MGC70870
ZDHHC18	LOC145783
ST14	RPGRIP1L
LOC729737	IPW
ADORA2A	TBX21
ORAI2	PDIK1L
CLRN1	FBXL2
DCST2	RAB6B

GRINA	FRMD3
SECTM1	STAT4
RENBP	NOG
AQP4-AS1	PLOD2
LINC00327	SENP8
TFEB	VSIG2
LPAR2	DENND2C
LY6G6C	GZMB
IFITM1	PAWR
RAB3A	MBLAC2
LOC102724927	PKIA-AS1
PSTPIP1	FKBP1B
SCARF1	PLCE1
PROC	CADM2
LINC01021	SNCA
FAM129B	GPR183
LINC01502	ZNF257
LOC646736	OR7E2P
GDPD3	KIF21A
MIR6781	ZNF878
INSC	LOC100499484
PLOD1	NTN4
CSNK1D	MYO5B
TYROBP	ACSM1
DDAH2	SPEF2
CDH18	AGAP1
TEPP	PDZK1P1
NLRX1	MIS18A
IGFLR1	LCA5
MIR5685	RPL22
POLR2J	LIMA1
TMEM91	GZMA
GRN	GMPR
LINC01441	RPL9
TMEM176A	LOC101928696
SLC16A5	AKR1C4
LOC399715	LINC00540
RGS14	ZNF583
SLC22A18	PKIA
LINC01209	NDUFA5
GSG1L	FLJ27354
HCAR3	TMEM106C
PAFAH1B3	PDGFB
PGLYRP1	PCED1B
PLEKHM1P	TAS2R43
SPI1	EFCAB13
TNNI2	ESAM
MROH5	TBC1D31
RAB43	NUDT9P1
SBNO2	IGFBP4
HYAL2	ARHGAP5
TBKBP1	ZFP37
MUC5B	PLK4

RAPGEFL1	HEG1
TBX6	CCDC176
SLC26A6	LOC101928100
RHOG	KGFLP2
TKT	SPAG16
MYO16-AS1	SLC9B2
MYO7B	USP46-AS1
SH2B2	KLKB1
PLXNA2	GATM
USB1	LINC00987
ZFPL1	VEPH1
MIR4440	AK5
LOC101928052	LOC101927156
MYH7B	C12orf42
TULP2	KLF12
PRAM1	SLC38A11
LRRC25	DCLRE1A
PLSCR2	PARD3B
MIR4638	SMC2
CFP	LANCL3
TMEM132A	L3MBTL4
UNC93B1	ZNF57
SIRT7	CCDC34
LINC01002	LINC01151
TRPM2	FUT8
POR	XK
SFN	AXIN2
FFAR3	ENPP5
CHKB	ZFP82
MIR26B	ZNF599
LOC100288069	CDKL4
LRFN1	HRASLS
LOC101928530	TC2N
LRRN2	TXK
MFSD7	LINC00853
EMX1	ITM2A
MCOLN1	ZFAND1
LINC00106	TSPAN13
CYHR1	DEPTOR
IL1R2	RNFT2
TCIRG1	TOM1L1
EPHB2	RPL7
MCAM	FRG1CP
JAK3	RPS20
GPR132	KLRC1
DSG3	IQCH
SEMA6B	SLAMF1
ZNF787	KDELC1
TSSK6	ARL6
RPS6KA1	ST7
FCGRT	LARGE-AS1
PABPN1L	ACVR2A
ATHL1	SLC47A2

SLC4A9	NAP1L3
SEMA4B	KLRG1
DPEP3	OXTR
CCDC153	BLNK
TWF2	NFIB
GAS6-AS2	EFNB2
LOC100507175	CMC1
SRL	DCANP1
LOC101928847	TACR1
IL1R1	BCL2
MIR6891	BEX5
MICAL1	AKT3
BCKDHA	NEXN
S100P	DHRS3
AATK	MTRNR2L6
MIR141	LIPC
MRGPRX3	ZNF32-AS2
SH2D3C	ACVR1C
CLEC18B	WNT16
FLOT2	SIM2
TREML5P	SORBS2
KRBA1	TPD52
LOC101928674	NAP1L2
KRT13	NT5C1B
ZGPAT	IGFBP3
STAB1	TPTEP1
SH2D6	GUSBP2
WFDC5	SPATA9
NUDT22	EEF1A1
ABCA7	ENPP4
BAIAP2	CD3E
FAM89B	RPS3A
OPLAH	SPTLC3
ZBTB17	LOC400685
MXD3	TRIM10
MIR7848	CCDC89
FSTL3	HACD1
INPPL1	AGBL3
ACE2	E2F5
SEZ6	GCSH
ZNF341	LINC00161
CHMP1A	C3orf52
MAP1LC3A	ZNF610
SULT1A1	LANCL1-AS1
NFKB2	MANEA-AS1
DEF8	KLHL14
ELL	LOC101927412
MIR6131	NUAK1
GNG4	SH3BP4
FNTB	COLGALT2
GGT1	PCDHGA11
ASPRV1	A2MP1
ZC3H3	DLGAP1

SLC8B1	ABCC4
ACOT8	GPR89B
AURKAIP1	C6orf201
SYNGR2	PCSK6
RXRA	ACSBG1
CMTM1	C12orf79
SLC35G3	TMEM133
LRCH4	TUB
GNB2	TSPYL5
B3GALT4	RPL21
MIR29C	B3GAT1
TPI1P2	EOMES
CSF3R	MIPOL1
CES1	MAMDC2
EFNA1	LOC100506136
RARA	GTSCR1
FOLR3	LINC00534
RASL10B	SAMD3
VPS9D1-AS1	WFDC21P
GAA	HSF5
NCF1B	BFSP1
NFKBID	FTO-IT1
CYP4F12	UQCRBP1
FAM83A	A2M-AS1
INAFM1	GLYATL1
LTC4S	CENPV
ARMC7	LOC101928896
CTSD	ZNF442
PAQR4	FAM221A
SUSD2	MMP21
C20orf24	MACROD2
LINC00671	PZP
GNA15	IFNG-AS1
MIR4481	CXorf57
C11orf94	IL7
SSPO	RAPGEF5
ZNF670-ZNF695	LINC01389
ITGAX	MYOT
GALNT4	GPR174
ELF3	CNKSR3
PRSS36	LAPTM4B
EPHB3	PTPRD
LINGO3	DLG3
KCNK7	CTAGE7P
MPV17L2	CCDC102B
MBOAT7	SNORD91B
BTNL8	CTH
NFAM1	ARL10
MIR23A	PDE6C
SCAMP4	LOC100996579
ZSWIM8	MIR548AR
ALDH3B1	DPH6
FLJ41200	ZNF571-AS1

COL18A1
MTG2
MIR3605
TMPRSS7
MIR8085
PNPLA2
DGAT1
HTRA1
TYMP
ASGR1
MIR4296
ZNF775
B4GALT2
CDC34
KDM4B
SPACA6P
MYO1F
CLASRP
RRP12
MIR1250
ACAP1
DBNL
NCLN
CASC9
PGPEP1L
RASGRP4
BLVRB
SCYL1
MMP14
FBRS
ADAM8
RBM42
SH3GL3
TNFAIP6
ZNF608
GABRR2
ABCD1
CASP5
MIR4312
LINC00999
PTK6
PRDX5
LIMS2
ZP3
LLPH-AS1
PLEKHM1
SOWAHD
ICAM3
LPPR2
GTF2IP20
MIR4313
HPX
RAB20
PCDH17
TRPC6
IL2RB
BCL11B
TWSG1
IL24
PTPRG
FAM150B
OSTCP1
RPL39
ARHGAP20
PCDHGA10
HMGN3-AS1
ZNF391
AFAP1
MEST
SYNM
NSG1
ZNF660
ZNF781
LRRC69
CCDC168
ZNF287
KIAA1524
PHOSPHO2
PTPN20
GCSAML
CHIC1
THRA1/BTR
CD2
ZNF880
TRPA1
S1PR5
MINPP1
ANKRD31
TSGA10
LOC101928767
LOC100507346
NAALAD2
LOC100996286
ANGPTL3
MIR4461
FCRLA
SNX24
RET
GAS1
UBAC2-AS1
SLC35F3
COL5A2
THBS1
OSBPL10
TDRD15
INPP4B

RABAC1	LINC00892
MIR30C1	DYNC1I1
NATD1	HKDC1
SLC28A2	REREP3
MIR6753	SMAD5-AS1
CEP295NL	ELOVL6
TMEM86B	GRAMD3
C17orf62	FECH
TRIOBP	GRK4
CCDC9	MLF1
EFHD2	SGCE
GSDMD	LOC101928794
PPP1R32	THEMIS
WDR13	EID2B
HDAC7	PDC
VSIG8	TTC21B-AS1
OR52W1	TAS2R19
ADAP1	ACKR4
HDAC5	LRRC39
WFDC12	DOCK9-AS1
CYP27A1	A2M
FAM166A	GAS2
ASAP1-IT1	DKK3
FLII	SERPINE2
SLC29A4	RPS23
LMAN2	GZMK
GALK1	MEIS1-AS2
SH3RF3	KLRC4
SPINT1	CATSPERB
PREX1	TIGIT
LOC728175	FAM171A1
CTRC	NUDT10
DOK4	IKZF2
TYK2	ANKRD20A8P
ARHGAP26-AS1	ZNF32-AS1
LOC100507437	TMOD1
C10orf54	SPATA13-AS1
LSP1	SYN3
ARID5A	SNORD105B
LOC102723354	FGFBP3
SLC43A2	ITFG1-AS1
NBEAL2	ZNF502
CCDC88B	KIAA2022
P2RX1	HRAT92
MIR3175	RPS27A
S1PR4	SERPINI1
C20orf195	TREML1
EFEMP2	LINC01184
RUSC2	LIX1
MIR205HG	PERP
COL23A1	NUF2
LOC101928597	LPAR4
LINC01001	CAPN14

FRMD1	STAP1
WASH3P	XIRP2
DND1	PAX5
GALE	ADGRB2
KRT8P41	NDFIP2
ITIH1	C11orf1
BRMS1	TRHDE
SART1	PWAR5
MIR373	ANKRD20A12P
APOBR	SYBU
IL17REL	C8orf46
ABTB1	UMODL1
DNM2	TSPAN33
MIR646	LOC152225
UNC13D	FAM171B
SAP25	PRH2
PQLC1	MIR553
MAP2K2	CD1C
TRAPPC5	ELOVL4
LRP3	ADGRA3
PGLS	SNORD69
RNF31	ZNF285
MARCO	MLLT10P1
TMEM150B	EFCAB6
DUSP13	KCNC1
FCN1	RTKN
PANX2	MICU3
JUNB	GLT8D2
KIF25-AS1	MIR155HG
PDLIM4	LAIR2
ZMAT5	TH2LCRR
ZBTB48	GLIDR
TMC4	RNF182
TMEM259	DIRC3
BAD	MRPS33
SLC27A1	SLC4A10
NANOS3	ZNF233
CDH23	LOC102723885
CERS4	CHRM3-AS2
LINC01271	SGCD
MOXD2P	MEOX1
CFAP45	SYTL4
SPATA2L	CD72
ITGA5	SNORD116-18
ARHGAP27	SRD5A3-AS1
TLR9	SLC16A14
MIR6819	KIAA1324L
LYPLA2	SNORD116-24
FOSL2	MAP2
CNN2	LINC00494
NOD2	BEND5
ICAM5	C12orf29
PI3	AMOT

MIR1273A	ISPD-AS1
HPCAL1	SLC9B1
GRK6	NUDT11
TEX22	C12orf45
FAM98C	HLF
ESRRA	MAP1A
ITGB2	LGR6
RAB40C	NEFL
LAMTOR4	EPDR1
MMP24-AS1	SYTL2
TCEB2	MC5R
C11orf98	STEAP1B
TNNT3	MIR548P
LINC00937	MCOLN3
KCNS2	CLECL1
MIR657	ZNF300
MROH6	TMEM45A
RBP5	ECM2
RNF40	ANKRD18DP
PVRL1	PTPRF
CD300LD	MYL9
PNMAL1	DPP4
SH3RF3-AS1	EIF5AL1
UNQ6494	ACSM3
MED16	GCSAM
ISYNA1	GTSF1
RHOT2	PRSS35
KRT23	CXCL3
LRRC29	TRIM2
GLIS2	SORCS3
IFITM5	CYP7B1
RILP	UGT2B11
RIN1	KLHL4
CNGA4	MIR4458HG
PAQR6	KLHDC1
ABCA13	FLJ37201
TOR4A	AKAP7
ZNF219	TTC3P1
TMEM176B	TUBA8
ANKRD35	FAM83D
ZNF296	TPH1
ZDHHC12	OST4
CST7	TSPAN5
SIGLEC5	SNORD111
MIR4736	GTF3C2-AS1
MIR3939	TARP
COMT	PCDH9
FCHO1	RNU6-57P
MAFK	GYPB
BEAN1-AS1	OR52N4
PTOV1	LYG2
MIR6075	OCM
VPS18	CFH

GRB10	ZNF208
PARP10	FAM218A
ABHD8	MTCL1
TMEM8A	C10orf35
LINC00266-3	MTHFD2L
UBE2S	PPP1R17
ZYX	BDNF
TMEM115	PVRL3
COL9A3	ARHGEF38
VNN1	RCAN3AS
MMP25-AS1	DYRK3
BAIAP3	ZNF711
A1BG-AS1	DEPDC4
RETN	CD160
COL11A2	RFESD
NDUFA11	RAB3C
APBA3	RBPMS2
AMDHD2	SLC17A8
C9orf106	LINC01088
G6PD	MAGI2
MCCD1	NPR3
DEAF1	MYEOV
ADCK4	SNORD116-15
TGFA-IT1	PTPN13
PNKP	GYPA
EHD1	NAT8B
MIR497HG	CAPN11
MIR191	DPY19L2
MAP2K3	RLN1
SNAI3	HS3ST1
MUC1	HNRNPA1P10
SELPLG	STRADB
TNFRSF12A	LINC01291
FGD3	OLFM1
KLF16	LINC00612
CD177	FZD6
NUDT18	ERC2
ZNF408	LOC105375734
PSORS1C3	SPON2
LOC101927153	WEE2-AS1
DNAJC4	PODN
CYBA	KLHL13
LOC101928977	SUCNR1
TESC	CAMK2N1
CCS	FAM227B
LGALS1	DNASE1L3
CPNE9	TAS2R13
KCNH3	LINC00515
PEX16	RPL26
FLJ21408	PCDHGB6
C16orf86	MAP9
GPBAR1	ASPN
TFPT	RORC

NR1I3	DNAJC6
TXNDC2	LOC101927391
PTPN6	LOC100132111
ODF3B	USP51
TCN2	PLSCR4
LGALS8-AS1	SCARNA27
ACTN4	TRPC1
CAPG	ACCSL
C11orf42	LOC100289561
TNIP1	CDR2L
SH3GL1	CASC15
DPM3	TOLLIP-AS1
XKR7	ANTXR1
MYLPF	S100B
HLX	MLC1
ROGDI	ACOT4
MYBPH	ROR1
C9orf89	SPAG17
CLEC5A	TMEM182
GADL1	RPS27
CTDSP1	RPSAP58
FAM209B	INTU
MIR101-1	STX18-IT1
WBP1	ACTRT3
FAM20C	MALRD1
METRNL	LOC101927179
CIRBP-AS1	KCNH8
APC2	UBE2Q2P2
SHARPIN	C17orf78
PCP2	HOMER2
C16orf93	LY6G6E
RAPSN	TRAT1
TNK2	ADAM23
MIR6807	LINC00925
EXOSC5	TRHDE-AS1
DAPK3	LRRC66
MYO7A	PLA2G4C
TSC22D4	PSMA8
BRI3BP	THCAT158
RAPGEF3	HUS1B
SELO	COL4A3
OGFR	LOC101928605
MAP1S	ZC2HC1A
MGC16025	NPY6R
LOC101927795	MTUS1
CDC42EP2	LINC01036
R3HDM4	RNU6-19P
CDC42BPG	SCN3A
PRKCDBP	RNU4-2
PDLIM2	GLI3
COPE	DNAH12
WASH2P	GYPE
GK3P	FAM186A

MAP3K11	C9orf153
FZR1	CEP112
SH3PXD2B	MMP1
NDUFA3	CENPK
DGCR9	DDX11L10
TIMM17B	TAS2R4
DCXR	LINC01535
HAAO	LEFTY1
TNFRSF1B	LRRC32
HSPA7	IL17RE
RFXANK	TSPAN9
RHPN1	ANKRD18B
LINC00266-1	OR2A4
ARHGEF17	ZNF750
ADGRE5	ANK2
SRPK3	GPR19
RHBDL1	ADORA3
LINC01490	SPDYE3
PRKD2	MTRNR2L9
FERMT3	COPZ2
UBXN11	ARHGAP42
UPK3A	AKR1C2
SEPT5-GP1BB	VIL1
ZNF593	HMGN5
ZNF414	MTRNR2L2
BATF	HOXA10
FAM106B	AFAP1-AS1
OXER1	NPPA-AS1
NCKAP5L	KLRK1
ARPC1B	HECTD2
FBXL19	ZNF229
MYO15B	SLC35G1
GSN	PCDHGA9
PPP2R3B	TMEM14A
SEC14L3	KIF18A
NUDT14	GLYATL2
CRACR2B	SNORD14B
PCAT7	WDR63
KCNE5	BEND4
EIF4EBP1	LOC389831
LINC00607	SNORD110
FAM3A	WNT2B
MIR5187	LOC442028
CAPN1	ENTPD3
SPSB3	KLRAP1
HELZ2	SNORD87
METTL7B	CRYZ
SIRT6	ZNF311
MTHFS	C2orf54
MAST3	GOLGA8O
HPYR1	ABCC3
SEMA3E	CDKL3
USP29	MAD2L1

ABCA2	ZNF462
KDM6B	GCNT4
MIR6769A	LINC00669
LMNTD2	VIT
MIR140	DNAJC28
GCAT	LDLRAD4-AS1
ACAP3	WBP5
CATIP	LOC101929555
BSG	KLRC4-KLRK1
MAP3K15	LOC101927620
TMED1	CD1E
ST6GALNAC3	TGFB2
TEN1-CDK3	LAMP5
MED25	TXNRD3
ARG1	FGF9
RBCK1	ALS2CR11
RPUSD1	MAFIP
TSSC4	SPOCD1
PLEKHM2	RAB38
ECHDC3	BTNL9
LINC00536	HFM1
POLR2E	CD9
RIN3	EMBP1
SIPA1	CRYM
HCG4B	LINC00544
CDHR5	ENAM
SLC38A10	LEKR1
PLEC	HLA-DPB2
MPST	METTL21C
VWA7	POF1B
PLEKHG6	COL5A1
SLC52A2	SLC26A3
ATG4D	LOC644554
SDF4	LRIT3
CHIT1	KLRC3
RN7SL1	LOC101927438
SSNA1	PRF1
MIR1273D	LOC101927482
LOC344887	LDB2
MYO16	TRPV3
HP	GBP1P1
GPR17	GPC5
RNU86	HEPHL1
LYL1	CCDC38
GPR137	CCDC150
CARD9	ZNF705E
FBXL6	STAC
ARHGAP4	LINC01372
NAPRT	ZNF157
RNASEK-C17orf49	RNVU1-15
VPS28	NUP62CL
LOC388282	STYK1
LOC100131635	KCNIP4-IT1

TCAP
EIF4EBP3
PRR25
PITPNM1
TREX1
ABHD16B
IL17RC
RCOR2
RHBDF2
PSD
PLK3
PADI2
MIR7109
PIN1
GPR84
CLVS1
GMIP
RTEL1
WAS
ADRBK1
TNFRSF4
PPP1R12C
BEAN1
TEX28
FLJ20021
NDUFA13
MIR6876
EME2
TNFAIP2
CCDC154
ALDOA
LINC01127
TSR3
C19orf24
MAPK11
OSGIN1
NLRP6
MVB12A
C19orf25
FAM65A
CORO1B
CHST13
AGRN
MIB2
NR1H2
MLST8
CORO1A
SNORA76C
LYPD8
SNORA80B
ARHGEF1
ADRM1
JOSD2
OPA1-AS1
ABCA8
GOLGA8N
TMEM56-RWDD3
C1QL3
FAM19A1
NETO1
TNFRSF13C
BCORP1
ARHGEF28
TFCP2L1
NELL2
LINC00943
CCL4L2
PCDH1
SNORA26
KLRC2
CA1
PTCRA
MTRNR2L8
SNORD97
RNY1

TBC1D10C
LOC728752
TSPAN4
ZNF213
SULT1A2
XAB2
PIGQ
TNRC18
ASL
SLC9A1
RNPEPL1
LIMD2
OLAH
TAS2R40
TELO2
FKBP8
LRRN1
MAMDC4
CHRM3
E4F1
LRP1
TRIM9
KAZN
ANKRD22
ZNF385A
LMF2
C19orf35
MBD6
NOTCH1
ORM1
KIF21B
HMHA1
CIC
MOB3A
SLC2A6
FMNL1
INTS1
RN7SL2
ISG15
MGRN1
ARAP1
AIP
FURIN
SYTL1
MAPK8IP3
RPPH1

GSE45376 SCI vs. Sham group

Up-regulated gene	Down-regulated gene
CDC45	NALCN
H19	BTBD17
KLF6	GLRA1
TBX2	EGFL6
NGFR	CTTNBP2
WNT9A	GABRA2
SLC22A18	KCNN3
CCND2	BCL11A
SLFN4	WFDC18
TH	SLC26A3
TSPAN32	GABRG1
TRIM25	FNDC5
SCPEP1	MNX1
ITGB2	FOLH1
CDH1	GRIA3
BCL6B	RMND5A
CLEC10A	PEG3
MX1	ZIM1
FAP	REC8
ACVRL1	BRAF
ITGA5	ABHD3
ADORA3	GRIN2D
TMIGD3	PPP1R17
SOX9	PLAG1
HK2	CCDC65
CD52	PHYHIP
ABHD15	LIN7B
LOXL3	HLF
DNMT3L	GRM3
SULT5A1	CACNA1E
RPL13	KIFC2
SERPINF1	BCAN
TCF7	SLC1A2
DBH	PRLR
MMP11	FAM131C
MMP14	SLC34A3
SLC7A7	ITIH3
CCL3	ATP7B
S100A4	RUNX1T1
S100A6	MC5R
ICAM2	HAPLN4
NHP2	GABRB2
KCTD10	1700008O03RIK
COL6A1	GPRC5B
LGALS9	FAM163B
CFP	TRPM2
TIMP1	SYN2
UXT	TEX15
UHRF1	TEX11
RAMP2	POU6F2
LSR	SLC38A3

ITGB7	CACNA2D2
RARG	EBF3
ACP5	GABRA1
CNN1	GRID2IP
UBE2C	CFAP161
TMEM79	VIPR2
COL18A1	CAMTA1
TUBB6	ENTPD2
PAX9	MAP2
COL1A1	XK
FOXM1	FAM184B
TUBB5	TENM1
FKBP10	HSD11B1
ACAP1	CACNB4
IFRD1	PLXDC1
CLDN15	SLC12A5
IL16	TRIM37
TCIRG1	GLRA2
NAGLU	GLRA4
CAPNS1	CACNG2
LTBP1	ETNPPL
SLC1A5	GDPD2
VWF	TLCD1
ESAM	GRM1
SSR4	FAM184A
LTBP2	MGAT4C
NR1H3	LRRIQ1
SPI1	SLC6A15
STAT6	LIN7A
NAPSA	CFAP54
PPM1J	LGR5
RHOC	APPL2
TEAD3	NSG2
DEF6	FGF22
ANGPTL4	GABRG2
DBF4	MATN3
IRF9	NTSR2
DHRS1	UBXN2A
RGS19	PFN4
RGS20	RAB37
SCIN	GRIN2C
AXL	ASPA
TGFB1	CAMKK1
SHH	CORO6
DENND1C	SPAG9
LCP2	CACNA1G
PLIN4	GUCY2E
CHAF1A	ALOX8
PLA1A	MYH10
MCM2	CDKL1
ADGRE5	NOVA1
IL17RA	AKAP5
LAMB1	SPTB

CD36	LRRC9
BCAM	CLMN
RELB	4930447C04RIK
KLF4	RPS6KA5
BCL2L12	CCDC88C
HCK	UNC79
MOB3A	ASB2
FKBP11	RGS6
RPS11	DPF3
INMT	FAM161B
CYP4F18	ESRRB
IER3	MEG3
FOSB	EXOC3L4
ERCC1	HECW1
CP	RYR2
HAS1	MAK
ITPKC	ID4
COQ8B	1700001L19RIK
KIF20A	HAPLN1
DNASE2A	OTP
GM38426	RAB3C
CALR	MTX3
BAX	FGF10
IL7R	NR1D2
RPL8	THRB
STAT3	LDB3
STAT5A	SLC35F4
CAVIN1	DNAH12
COL5A3	CHAT
PPAN	FGF9
ANGPTL2	AMER2
PTPN6	CACNA2D3
LPCAT3	CHRNA2
IL11	KLHL1
RALB	GPC5
DNAJB11	CLDN10
CTSE	JPH4
ETFB	CIDEB
AQP1	SKOR1
CNN2	DNAH5
LY9	ZFPM2
CD244A	CSMD3
ADGRE1	CDH10
CCL24	FBXO32
GRAP	LRRC6
PLOD3	TTLL8
MAPK13	CACNA1I
CRABP2	ENPP2
NES	ADAMTS20
HSPB1	SEPTIN3
CSTB	SHISA9
SH2B2	FGF12
CD44	CEP97

WDR1	UBE2V2
SLC2A9	SIDT1
CCN4	LRRC74B
KLF5	NCAM2
SPC25	AIFM3
FGFR4	CFAP91
NID1	STXBP5L
MCM5	CHODL
HMOX1	DGKG
IL27RA	D16ERTD472E
ASF1B	ROBO1
DDX39A	SLC15A2
POR	ILDR1
AMPD3	SCN8A
MMP8	LRRC71
TNFSF14	GABRR2
GATA6	PRKN
TRPV1	SLC25A27
RCN1	SLC5A7
TYRP1	ENPP5
PRG4	KIF6
UPK3BL	GLP1R
FBLIM1	EPB41L3
HSPB7	PJA2
TEP1	NRXN1
ARPC2	PREPL
RPS9	ST6GAL2
CRIP2	GRM8
CRIP1	COL11A2
FBLN1	PSD2
ELOVL1	IMPACT
CDC20	MEGF10
SRM	HTR7
EPHA2	ASAH2
PTBP1	PELI3
CYBA	SMARCA2
SLC4A1	TDRD1
CDT1	MYADML2
APRT	FN3K
GMNN	CNNM1
CDK4	SEMA4G
METTL1	SACM1L
DDAH2	ZFHX4
CLIC1	SP4
LSM2	TAC2
POLE	USP33
DENND2C	ZFP711
CAV1	PHKG1
BCL2L1	FGF14
TWIST2	NAT1
RHOA	KLHL4
ALDH16A1	ARHGEF9
ID3	CNKSR2

CRLF1	RPS6KA6
CTSD	TMEM47
RPLP1	A930017K11RIK
PHOX2A	HSPA4L
FGFRL1	D3ERTD751E
FHL2	GDAP1
SERTAD1	CLEC3B
ELK3	ST8SIA2
ARPC5	DHTKD1
MGST1	MYO5B
RPS18	GRIA4
RPL10	OPRK1
CD163	SNTG1
BMP7	TFAP2B
DYNLL1	PTH2R
TBX1	ICA1L
CCL8	KHDRBS2
TRPM5	CRACDL
RARRES2	COL19A1
PTTG1IP	B3GAT2
UBE2G2	SPHKAP
MPO	CYP27A1
LPO	TMEM169
MET	SLC16A14
KCNQ1	EPHA4
FPGS	NPPC
APOBEC3	IQCA
OIT3	CDH7
FXYD5	LYPD1
NUAK2	PTPN4
RPS25	SYT2
HYAL2	LHX4
IFRD2	KIF26B
RASSF1	EFCAB2
SLC3A2	ATP1B1
SLC47A1	ESRRG
TNFRSF13B	SLC30A10
IFI35	UCMA
MRPL52	MYOC
WNT2	LYPD6B
KDELR3	GAD2
MCOLN2	KCNJ3
AKT1S1	1700007K13RIK
TBRG1	UPP2
ODC1	FIBCD1
PABPC4	GCA
EXOC3L2	SLC4A10
CPB1	STRBP
SNAPC2	PAX8
SCARF2	HNMT
RPL15	DEPDC7
STEAP4	ELF5
KIF11	FAM227B

MLKL	SNAP25
RETN	ITPKA
RPS5	CHGB
AMOTL1	NAPB
ETV5	ZDBF2
ALDH1A2	EDN3
TNFAIP8L2	NTSR1
MCEMP1	NKAIN4
TRPV4	CHRNA4
BICC1	TNIK
PIEZO1	STOML3
CSF1	NBEA
STC1	KCNAB1
TMEM208	SERPINI1
SURF4	SYPL2
GALNS	SLC16A4
LRRK1	TMEM144
ALDH1A3	OLFM3
ACTN1	COL11A1
ABCA1	NDST4
GADD45B	GUCY1B1
SLAMF1	GLRB
ZDHHC12	TACR3
CYBB	LRRC7
CD48	CALB1
EGFL8	ALDOB
NOTCH4	TMEFF1
LPL	PTPRD
ATP6V0E	SH3GL2
STEAP1	DAB1
ANXA9	DNAJC6
PLEKHO1	CALR4
NFE2L2	MYCL
FCRLS	EPHA8
CD5L	ATPAF1
NCAPG	HTR6
FCGR1	VWA5B1
NCF1	EXTL1
LBP	EPHA10
FLI1	CDK14
CTSZ	TAS1R1
COL20A1	PER3
PPFIBP1	GABRD
CD34	PROM1
CD274	PCDH7
IL19	RNF32
MAPKAPK2	PPARGC1A
IL10	GABRA4
H3F3B	GABRB1
BIK	SLC10A4
SULF1	NMU
SLPI	EPHA5
SDC4	SPARCL1

IL13RA1
BRCA1
GSDMA
RPL19
C1QTNF1
IGFBP4
CDC6
TNS4
TNFAIP1
CD40
ADA
BIRC5
MMP9
GHDC
SLC12A7
SLC12A4
MYO1C
DHX58
NKIRAS2
MYBL2
PTGIS
CYTH4
CYB5R3
MAFK
MFNG
PMP22
PFN1
GPX3
KPNA2
ABI3
TRPV2
GABARAP
TBX3
IKZF1
COPZ2
CD68
LSP1
IRF1
CXCL16
MED11
CCL6
CCL4
MAP2K3
E2F2
RAB3D
DNASE1L1
CCL9
ISYNA1
RAB5C
H13
CHTF18
AHR
DPEP1
CDS1
TESC
RASSF6
AJM1
RIMBP2
CIT
UNCX
RPH3A
WASF3
GLCCI1
AASS
SSPO
HERC3
CCDC184
NAT8
CNTN6
GRIN2B
RERG
LMO3
SLCO1C1
SLCO1A4
CPNE9
TTLL3
ST8SIA1
ATP2B2
SLC6A11
PRMT8
PSD3
SLC7A10
SLC17A6
RAB30
ZKSCAN2
SLC5A11
FGFR2
VWA3A
CNGA4
ACSM5
D7ERTD443E
TRIM66
IGSF1
MCF2
AFF2
HEPH
GPR165
XKRX
DCX
CDKL5
RS1
CHIC1
GPM6B
GABRA3
GABRQ
ZFP92

NOP9	ATP2B3
PSMC3IP	TCEAL6
AOC3	PLP1
GIPC1	ADPRHL1
PLSCR3	GPM6A
TRIP10	NR3C2
RCN3	CBLN1
PDK4	CNTNAP4
MYDGF	DRC7
MICAL1	NECAB2
RAB32	PGR
SLC16A10	CAR7
LAMA4	TSNAXIP1
TNFAIP3	AGT
CRYBG1	PDGFD
DCN	DIXDC1
CDK1	DDX25
DUSP6	TMEM266
KITL	CAR12
SGK1	RWDD2A
ARG1	TDGF1
NEDD1	LRRC2
MTFR2	TTC21A
CCN2	HHATL
MOXD1	CCK
IFNGR1	THSD7A
HAL	CREBL2
SNRPF	6820408C15RIK
DRAM1	IGDCC4
DDX21	PPP1R9A
SRGN	CCDC85A
SGPL1	ACSL3
PLEK	SGPP2
2310011J03RIK	CAMKV
DOCK2	RASGRP2
KCNMB1	MYCBP2
E2F7	ASIC4
CSRP2	CLASP2
PHLDA1	ADGRB3
WIF1	CASKIN1
IRAK3	NAT8F2
COL6A2	GABRB3
TXNRD1	ST18
SLC36A2	NPBWR1
RHBDF1	ASB13
FSTL3	B3GALT2
HMMR	MAP9
FLT4	CCP110
HNRNPAB	GUCY1A1
GFPT2	CDKL4
RACK1	GRIA2
RASGEF1C	NEU4
PDLIM4	ZFP551

HAVCR2	FSTL5
UPP1	TSPOAP1
BTG2	EVPL
IGFBP3	CCDC15
MYO1G	TRIM59
SMTN	KCNH5
RPS27A	EDIL3
EFEMP1	FRAS1
XBP1	PABPC5
PRR11	PCDH11X
SHMT1	ASB16
PDIA6	TLE2
PIK3CG	PYGO1
SDC1	MDGA2
RSAD2	ZC3H12B
RRM2	ARHGAP5
CCL11	LCAT
MRC2	SLC25A21
PECAM1	PLK5
JPT1	FBXL21
GALK1	CCDC180
TRIM47	L3MBTL1
LLGL2	KCNH8
TXNDC17	KY
RHBDF2	LRFN5
PIMREG	KCNC2
NOS2	GRIN3B
SLC6A4	KRT222
NME2	CDHR3
ABCC3	SYT1
LRRC59	RNASE1
AURKB	DCDC2A
PIK3R5	ALDH5A1
NTN1	NME5
KRT19	PRTG
TOP2A	DGKB
MAP3K14	SLC26A8
POLE2	ARMH4
NFKBIA	FSTL4
PYGL	SLC2A13
BDKRB2	P2RY12
SERPINA3N	GPR101
ZFP36L1	TMEM255A
SUSD6	RNF13
CDCA7L	CDH8
EFCAB11	ETL4
FBLN5	CCDC148
LGMN	ZCCHC12
AKR1C13	TTLL7
FOS	KLHL13
TNFAIP2	FAM135B
GPR132	DPP10
SFRP4	ABCC12

IRF4	KIRREL2
GCNT2	SLC39A12
EDN1	WNT8B
SUSD3	TRIM17
RIPK1	DENND11
PXDC1	DZANK1
LY86	SLC9A7
GADD45G	RIMS2
FBP2	ZMAT4
SYK	RSKR
CTSL	RGS22
MXD3	FAM13A
PDLIM7	PPM1K
2210016F16RIK	INPP4B
GOLM1	6430571L13RIK
SLC6A19	NEUROD6
TRIP13	WNK2
CD180	SLC24A2
F2RL2	RAPGEFL1
IQGAP2	KCNJ9
F2RL1	DLEC1
THBS4	FAM126B
CENPK	GASK1A
IL6ST	NEUROD2
GPX8	GLRA3
FST	TCTE2
CDHR1	ANKRD35
NID2	TMC3
ANXA7	SRD5A2
PLAU	CIART
VCL	SLC35F1
ANXA11	ZKSCAN16
RNASE4	TRHR
ARF4	LMX1B
GPR65	BIRC7
GALNT15	HRH3
SEMA3G	HTR5A
ITIH4	WHRN
CTSB	CAMK1D
PRKCD	LGI2
ANXA8	MINAR1
SKA3	WDR17
ESD	CNTNAP2
LCP1	MICU3
EPSTI1	CNTN5
TNFSF11	CCDC27
RGCC	SGCZ
DIAPH3	CFAP70
SCARA5	RSPH4A
PBK	CCSER1
ESCO2	GRIN3A
CLU	TRMT9B
TNFRSF10B	STRIP2

BIN3	CPEB3
SLC39A14	RALYL
BMP1	SLC6A5
DOK2	DNAJC28
SPRY2	GABBR2
CLN5	TXLNB
ACOD1	GPR37
GDNF	PAK5
OSMR	PLCB4
FYB	NEGR1
DAB2	GJB6
LRP10	GPR26
HAUS4	ABCC8
AJUBA	TDRD6
SLC7A8	MAOB
FBXO4	ANKRD6
PSME1	DAAM2
TGM1	DNM3
ADCY4	TRIM7
RIPK3	WDR47
ANKRD33B	CDH18
RAI14	CDH12
PABPC1	ADAM22
SHCBP1	PPIP5K2
MATN2	SLCO4C1
MYC	CYP2S1
ATAD2	KCNA2
HAS2	MYH6
COL14A1	TTC3
SLA	CATSPERD
WNT7B	PLEKHH2
GTSE1	REPS2
FAM83F	PNMA8A
DSCC1	DNAH7B
PARVB	WNK3
PARVG	DMXL2
C1QTNF6	AK7
MYH9	SHTN1
SLC38A4	NINJ2
COL2A1	HTR2C
NCKAP1L	PAQR6
EMP2	GPR12
CLDN1	PTCHD1
MEFV	FMOD
EEF2KMT	L3MBTL4
APOD	GUCY1A2
GSDMD	RRAGB
GPIHBP1	BBOX1
LY6C2	RIMS1
LY6E	BC006965
LMF2	TRPC5
RETNLG	2700046A07RIK
CCDC80	FAM169A

CD200R1	NOL4
MCM4	ELMOD1
B3GNT5	MYO3B
RIOX2	DAO
TMEM45A	DZIP1
SDF2L1	BEND6
UMPS	NXT2
FSTL1	FREM3
HCLS1	OSBPL6
MYLK	SDHAF3
PDIA5	KLHL14
BTG3	PWWP3B
KNG1	NEK10
SAMSN1	JPH1
ADIPOQ	MRAP2
ADAMTS1	FAM166B
ADAMTS5	HECW2
CD86	FGF11
STFA2	NREP
PARP9	GM14117
PROS1	GREB1L
RIPPLY3	D830039M14RIK
CHAF1B	AQP6
CBR3	TMEM212
RCAN1	4930426D05RIK
RUNX1	GPRASP1
IL10RB	SPTSSB
IFNAR2	RAB9B
MIS18A	ADAMTS3
LALBA	KCNS3
DHH	TMEM145
GPD1	GLS2
LIMA1	GPR22
ATF1	EML6
CELA1	NKRF
NR4A1	SPINK10
KRT18	CNR1
SOAT2	ZFP879
IGFBP6	SNHG11
SNCG	SOWAHA
CDKN1A	BC024139
CXCL13	CXXC4
VWA5A	LINGO4
SERPING1	CSRNP3
SERINC2	ZFP612
KCNK5	FADS6
PARP3	D630023F18RIK
CRELD2	UCN3
TRIP6	PRRT3
CLEC4N	GM4779
TMEM176A	ANKRD34B
NFATC4	SHROOM2
PRPH	KCNG4

CDCA3	ADRA2C
RSPH3B	GPRIN3
THBS2	FRRS1L
SMOC2	OLIG3
MMP25	CHRM2
TNFRSF12A	VWC2L
SGO1	FAM216B
SLC29A1	NPAS4
PTK7	MCMDC2
BYSL	NXPH1
TREM2	GSG1L
PI16	PNMA3
PIM1	STXBP6
SIK1	PPM1E
ARHGAP28	NEXMIF
MYL12A	SCN4B
EMILIN2	KCTD4
NDC80	AR
XDH	SPIN2C
MAN2A1	TTC34
CYP1B1	SLITRK4
MCFD2	EPM2AIP1
NME4	LRRC75B
DUSP1	CFAP65
FKBP5	TET1
MAP3K8	CHST9
SVIL	DYNC2H1
ADCYAP1	GPR15
MYO1F	ZFP286
TAPBP	LANCL3
WDR46	ODF3B
PSMB8	GPR68
TAP2	CMYA5
BTNL2	ZFP30
STING1	KLHL34
GFRA3	DLGAP2
C2	RNF152
TNF	MROH7
PCDH12	MAFA
ARAP3	ANKRD34C
LVRN	D930020B18RIK
HBEGF	FBXO40
MBD2	ANKFN1
PMAIP1	FLRT1
LOX	DIRAS2
PPIC	SSTR2
IL17B	SOX1OT
LMNB1	KCNA1
RPS14	DIPK1C
CD74	PRRG1
PDGFRB	HES5
CNDP2	TMEM196
ANXA1	TMEM229A

INCENP	CCDC187
RAB3IL1	NWD1
FADS3	SLITRK3
MS4A7	SCRT1
MS4A4C	MSX1
MS4A6B	ZDHHC22
MS4A4D	DUXBL1
MS4A6D	RTBDN
FAM111A	KLHL11
LPXN	LONRF2
OSTF1	VSTM2A
TRPM6	RIMKLA
MS4A8A	ATP13A5
CCDC86	NDNF
SLC15A3	ANKRD34A
EHD1	PLCXD3
FAS	FRMPD4
LIPA	LRP1B
CDCA5	TENM2
KIF20B	PLS1
ANKRD1	TCEAL1
FRMD8	ADAMTS16
BANF1	FZD9
TMEM134	TMIE
CDK2AP2	CFAP46
RIN1	GRM5
ALDH3B1	OMG
GAL	AGMO
EFEMP2	EIF5A2
CTSW	DSCAM
FOSL1	LYPD6
LRP5	SLC35D3
RELA	PCDH20
EHBP1L1	OPRD1
PLCB3	FAM228B
FERMT3	LRRC4C
HHEX	FAM78A
CEP55	CCDC121
PIK3AP1	TRHDE
ADD3	GM5084
XPNPEP1	CDH20
MSR1	DCAF12L2
CASP7	GJA1
AFAP1L2	KCNS2
GFRA1	CREG2
BNC1	ZFP455
P4HB	OLFML1
ARHGDIA	MBLAC2
ALYREF	GHSR
PYCR1	PLCB1
CENPX	NAT8F3
CBR2	KCNJ16
ARHGAP19	SIGLECH

SLC16A3	USP29
UBTD1	GM5124
LOXL4	MAP3K19
ERLIN1	DCUN1D4
NFKB2	XKR4
HEXA	SLC6A7
ALAS2	F730043M19RIK
DNASE1L3	SEMA5B
SAT1	GPR17
PADI4	DNAH3
KDM5C	DOC2A
ITGA7	TBC1D30
CD63	IL1RAPL1
MMP19	ACTN2
CDK2	TRPM3
RPS26	BMPR1B
PA2G4	A330008L17RIK
IL23A	ADARB2
MYO1A	DLG2
NAB2	ZMAT1
SHMT2	KCNT2
AVIL	DNAH6
ADAM8	SV2B
URAH	FANK1
IFITM1	PTPRT
IFITM3	SPAG16
SIGIRR	DAW1
IRF7	GM12592
TALDO1	ADAMTS18
PIDD1	ADAMTS19
RPLP2	HS6ST3
PNPLA2	STUM
CD151	ZFP14
TSPAN4	SPOCK3
GUSB	TPRKB
TK1	LRRC36
PODXL	CADPS
MAP3K7CL	VSNL1
SHISA5	KCNN2
PFKFB4	SLC8A1
COL7A1	PM20D2
ALOX5	SRSF12
NMB	ANKRD24
SEC11A	DEFB42
IL6	SMIM10L2A
TYMS	NYAP2
LY96	CNTN1
RPL14	SMYD1
CCR1	GABRA5
ITGB1	M5C1000I18RIK
PDIA4	FUT9
NOP16	NELL1
HK3	ATP10B

CASP12	PCDH9
CASP1	NAP1L5
RDH10	ANO5
TRAM1	EPM2A
EEF1B2	GUCY2G
ACADL	EPHA6
CDK15	UNC80
CASP8	TMEM191C
CFLAR	NAP1L3
COL5A2	ABCD2
COL3A1	CAR10
IL18RAP	KCNQ3
IL1R1	UTS2B
IL1R2	MED12L
STK17B	PTPRN2
STAT1	APOL8
PTPN18	SLC35F3
CTDSP1	ZFP607B
SLC11A1	SCN3A
CXCR2	RYR3
IGFBP5	RIT2
FN1	COLQ
BARD1	GNAI1
DES	LDOC1
SP100	A830018L16RIK
HTR2B	TMEM178B
GPR35	ANKRD29
PDCD1	ABAT
INPP5D	CAMK2B
SERPINB8	CACNB2
GPR39	ADAMTS17
MCM6	CHSY3
RGS1	GM5148
STEAP3	MMEL1
MARCO	GM10037
PTPRC	QRFPR
TNNI1	ZFP420
UBE2T	KCNJ14
RAB29	COL25A1
RGS16	C1QTNF3
LAMC1	KCNC1
LAMC2	GRIN2A
NCF2	IL1RAPL2
OPN3	NHS
IFI202B	SYN3
IFI211	GM7361
TAGLN2	DBP
SLAMF9	HYDIN
UCK2	NTNG1
F5	NTM
SELP	KIF27
SELL	CALN1
SELE	PANTR1

PRRX1	2310069G16RIK
CENPF	A930017M01RIK
NEK2	DCC
ATF3	PLEKHH1
TRAF5	LRRTM1
FAM107B	CTNNA3
FCGR2B	KCND2
MCM10	SLC4A4
DDR2	GALNT13
NUF2	LSAMP
PRDX6	AGBL4
CENPL	DNAI1
MRC1	KSR2
TNN	PCLO
RSU1	TMEM91
VIM	PPP1R1B
NEK6	KALRN
PKN3	PATJ
APBB1IP	CCDC62
NIBAN2	MYT1L
ENG	UNC13C
RALGDS	HS6ST2
LCN2	ERBB4
GBGT1	OPCML
CYTIP	TRANK1
COL5A1	RPP25
HSPA5	OTOF
PHF19	DGKK
GSN	KCNC3
STOM	CCDC177
LHX6	KCNMA1
IFIH1	SYT10
EGFL7	LUZP2
AGPAT2	LRRC43
CARD9	WSCD2
FCNA	PLPPR1
NMI	GNG8
UAP1L1	CRB1
PSD4	SLC22A8
IL1RN	LIN28B
DHRS9	NLGN1
PRG2	TRERF1
SLC43A3	IPCEF1
SLC43A1	CADM2
UBE2L6	CNTN4
TFPI	HHIP
ITGA6	SCN1A
FBN1	GM25129
SLC28A2	NRXN3
CREB3L1	ANKDD1A
PDIA3	HMCN1
HAO1	CNTNAP5B
JAG1	ASB18

EHD4	LGI1
NUSAP1	KRT77
DLL4	KLRI1
SPINT1	VXN
SIGLEC1	MKRN2OS
RAD51	SMIM45
KNL1	AGTR2
KNSTRN	MACROD2
PCNA	EFCC1
DUSP2	SLC4A5
PROM2	D430041D05RIK
MALL	GJD2
BUB1	EFCAB1
IL1B	CGN
SNRPB	DDX3Y
LPIN3	NXPH2
CD93	ADGRV1
GINS1	NKAIN2
ANGPT4	INSYN2B
SLC52A3	CCDC153
TPX2	TMEM132B
MYLK2	FV1
AURKA	VMN2R84
ZBP1	CNTNAP5A
PTPN1	PNMA8B
CAR13	ZSCAN18
HELZ2	ZFP804A
AHCY	GAD1
PROCR	1700086L19RIK
EIF6	ARMC2
RBL1	CFAP44
FAM83D	RIMBP3
CCN5	GM960
SLC2A10	CDR1OS
OCSTAMP	GPRASP2
ECT2	9330158H04RIK
CCNA2	CCDC160
SLC7A11	H2-BL
POSTN	CYP4F15
TM4SF1	DOK6
WWTR1	CPLANE2
SERP1	TTPA
PTX3	FBXW15
OLFML3	GM12522
NGF	ZFP109
PTGFRN	TNFAIP8L3
TBX15	GM10684
HMGCS2	FAT3
S100A11	GM10710
LCE1G	GM14296
SPRR2J-PS	GM10714
NPR1	KCNG1
RAB13	ANKUB1

NUP210L	MYH7B
TPM3	GM5535
IL6RA	4833422C13RIK
EFNA1	CHRM5
GASK1B	ANO3
HADH	DCDC5
MCUB	NXPE3
TLR2	FADS2B
CASP6	CCDC162
FGA	SCN2A
SNX7	GM11681
DKK2	SLITRK1
IFI44	IGHM
EFNA4	ERICH3
CKS1B	KLHDC7A
GBA	AI593442
LMNA	CFAP74
PMF1	CCDC24
IQGAP3	1810010H24RIK
NTRK1	EVI2A
PEAR1	PRKCG
ECM1	GM14412
CTSK	D130040H23RIK
DEPDC1A	ZAN
BCL10	FAM228A
CCN1	TEX52
RPS20	COL4A3
ATP6V0D2	NAT8F5
CLCA3A2	H2-Q1
GBP3	GM715
GBP2	NAP1L2
STRA6L	ZFP133-PS
ANP32B	GM14827
NANS	GM5577
COL15A1	A730046J19RIK
TNFSF8	SBK3
TNC	OIP5OS1
TXN1	MEIOSIN
SVEP1	GM12992
PAPPA	GRIN1OS
PTGR1	D030047H15RIK
PTBP3	1700003D09RIK
B4GALT1	AW822252
AQP7	GM15328
CD72	GM14169
CAR9	9430041J12RIK
TLN1	PCSK2OS1
GLIPR2	G630016G05RIK
BNC2	A330102I10RIK
PLIN2	GM14204
HACD4	GM13446
ARTN	GM15816
CDKN2C	B230206H07RIK

LAPTM5	GM13546
PDPN	4933431E20RIK
ORC1	GM16638
GPX7	PLCXD2
TNFRSF1B	GM15246
TNFRSF8	A930006I01RIK
CPT2	KANTR
YBX1	GM11266
P3H1	4930506C21RIK
KIF2C	GM16835
RAD54L	A530058N18RIK
CYP4B1	SLAIN1OS
STIL	OLFR287
PLA2G2E	CFAP97D2
DDOST	GM3667
HSPG2	KLHL33
TINAGL1	MYOCOS
AZIN2	GCNT4
AK2	CEROX1
RNF19B	SIAH3
A3GALT2	GM8094
SH3BGRL3	PEG10
GPN2	GM20646
CSF3R	SMIM17
MAP3K6	1500015L24RIK
CDCA8	MUC3A
FGR	POU3F2
RCC1	FAM177A
HES3	NIM1K
PGD	SOX1
UTS2	D830030K20RIK
TNFRSF9	SYNE1
ABCB1B	KCNJ11
CAR6	GM8104
NOS3	FAM181A
H6PD	GALNTL6
ANGPTL7	A330023F24RIK
NPPB	D930007P13RIK
NADK	2610316D01RIK
MXRA8	9330111N05RIK
TAS1R3	A330048O09RIK
BST1	1500026H17RIK
SORCS2	D030068K23RIK
HTRA3	GM26633
FOSL2	4933406C10RIK
EMILIN1	1700040D17RIK
PGM2	MIR124A-1HG
SLC35F6	4930578M01RIK
CENPA	A930012L18RIK
FAM114A1	GM17597
PI4K2B	GM2061
CCKAR	A330033J07RIK
RHOH	B230323A14RIK

TEC	A730056A06RIK
SPP1	MIAT
AFF1	PANCT2
SLC10A6	9630001P10RIK
PLAC8	CELRR
ANTXR2	TUNAR
RFC5	A330074K22RIK
CXCL5	GM27199
PPBP	1110015O18RIK
PF4	ZFP383
EREG	2900052N01RIK
AREG	1700047M11RIK
CXCL3	MIR124-2HG
CXCL1	6030407O03RIK
TMED2	GM29443
KNTC1	A230077H06RIK
CXCL9	IPW
ARPC3	1700063D05RIK
ANXA3	NSCME3L
ACADS	BC055402
TES	GM19461
TFEC	5033404E19RIK
SNX8	GM2464
OASL2	PCDHAC2
ACTB	PCDHA9
UNG	A330015K06RIK
DDX54	GM20743
OAS1B	A230001M10RIK
RPL6	6330410L21RIK
ARPC1B	LHFPL3
RASL11A	GM18290
COL1A2	C530044C16RIK
TFPI2	GM30731
ELN	4933427D14RIK
EZH2	GM7298
EPHB4	CEACAM-PS1
PCOLCE	PNMA8C
TSC22D4	FRMPD2
MCM7	GM31135
PON3	FAM81B
CALD1	GM39129
AKR1B8	GM18782
CALU	A230057D06RIK
IRF5	C030029H02RIK
FKBP9	GM6145
TMEM176B	GM45623
GPNMB	GM33148
OSBPL3	IGIP
ZC3HAV1	GM32122
TCAF2	APRT-PS
ZYX	BTBD8
CLEC5A	GM32014
TBXAS1	GM39377

ANXA4
ADAMTS9
ARHGAP25
RPN1
PROK2
GXYLT2
SEC61A1
ALDH1L1
TMEM43
EDEM1
USP18
SLC6A13
SLC6A12
MFAP5
GDF3
PLXND1
CLEC4E
CLEC4D
CLEC4A2
CLEC2D
OLR1
KLRA2
YBX3
ETV6
BCL2L14
EMP1
PLBD1
ART4
MGP
ERP27
ARHGDIB
DERA
IRAG2
CAMK1
CIDEC
IL17RC
LTBR
TNFRSF1A
CD9
RAD51AP1
TSPAN11
TEAD4
CLEC2I
VASP
PGLYRP1
LILRA6
RPL28
SIGLECE
FURIN
IQGAP1
SEMA4B
PLIN1
NR2F2
GM10635
A330049N07RIK
A830082N09RIK
A330076C08RIK
GM10432
GM10790
9330199G10RIK
G630093K05RIK
GM38593
GM18649
GM1043
9330117O12RIK

CTSC
NOX4
CD22
TYROBP
2200002D01RIK
PSMD8
PAK4
CALCA
PDE3B
QPRT
KIF22
MVP
KLK10
PPP4C
CORO1A
KLK7
NUPR1
RPS3
IL21R
DGAT2
IL4RA
NSMCE1
TGFB1I1
COX6A2
ITGAM
LYVE1
ITGAX
ADM
PYCARD
TEAD2
CD37
BCAT2
ITGAL
BAG3
PLK1
ILK
HPX
CRYM
TMEM159
TRIM30A
TRIM21
MKI67
SWAP70
AKIP1
MRGPRF
FADD
CTTN
SASH3
ELF4
PRICKLE3
PLP2
WAS
RBM3

RBM3-PS
EBP
SLC38A5
MSN
EFNB1
PBDC1
CENPI
BTK
COL4A6
IL13RA2
IL2RG
RPS4X
FLNA
GABRE
BGN
RENBP
F7
F10
ANGPT2
ADGRA2
EIF4EBP1
COL4A1
COL4A2
RAB20
PTPN7
DUSP4
GSR
RBPMS
SAP30
HPGD
CASP3
SNX20
HP
CES1F
MMP2
CENPN
MT2
MT1
SLC12A3
USB1
TPM4
B3GNT3
JAK3
PGLS
GINS2
CRISPLD2
COTL1
IFI30
LSM4
LPAR2
CDH5
CMTM3
CES2G

RRAD
CBFB
PSMB10
ADAMTS8
ST14
BIRC3
OAF
REXO2
NXPE2
ETS1
TIRAP
PPP2R1B
TAGLN
MPZL2
STT3A
SLC37A2
ROBO4
HMBS
KANK2
CCNB2
ANXA2
KIF23
NNMT
1700017B05RIK
PSTPIP1
STRA6
COL12A1
LOXL1
CGAS
TMED3
CTSH
ADAMTS7
PLSCR1
PLSCR2
PLOD2
CIAO2A
SNX1
RBPMS2
SPG21
SMAD3
TBX18
NT5E
CRTAP
CMTM7
TGFBR2
NGP
PTGS2
NRADD
LTF
TRIB1
STAC
MYD88
CSRNP1

RPSA
SLCO2A1
TRF
ACPP
MANF
MAPKAPK3
CISH
UBA7
SLC25A20
CHSY1
FHL3
OAS3
OAS2
NLRP3
TRIB3
MDFI
SBSPON
FOLR2
INPPL1
SLC8B1
TRPA1
TROAP
SRXN1
SLC10A3
ARAP1
P2RY2
STYK1
NUP93
FKBP1A
SLC16A8
NFATC1
CDO1
CALHM2
EIF3L
TRIOBP
NOL12
RNF125
SLC35D2
TIE1
AA467197
RAC2
NOC4L
LPP
TNXB
CHST2
RTP4
PUS7L
FRRS1
ADAMTS15
CRLF2
PRSS35
CDC14A
ASPRV1

CASP4
ARHGEF5
APOL6
IGF2BP2
UCP2
TLR13
DYSF
TPSB2
FGB
STON1
LGALS3BP
ASPM
SLC16A2
RFC3
SLC66A2
FANCD2
LYL1
SCN11A
VAV1
TSPAN8
CCND3
IRF2BPL
LOXL2
BATF
KIF4
BRIP1
PLCG2
CPNE2
AI661453
LIF
TBC1D10A
NEURL1B
PARP14
TMEM30B
EDA2R
IFIT1
SCARA3
UACA
GBX2
GJC1
SCN10A
PPP1R18
CHST11
LY6D
TIPARP
CD300LD
CD300A
GALP
ITGA2B
PPP1R1C
GRN
DUSP5
HROB

CD207
GPSM3
GNA15
PLVAP
CXCL10
LRR1
NCAPH
CDHR2
TMEM106A
CEBPA
VAT1
IGSF6
BAZ1A
CCL5
FAM167A
SHC4
LARS2
SLFN8
PKIG
HPSE
ROR1
CCL12
NFKBIZ
PARPBP
CCL7
CCL2
FIGNL1
TGFBI
IGFBPL1
MIDN
SBNO2
TNFSF9
MELK
ISG15
ARHGAP45
ALG8
DOK3
GGTA1
ACTA2
TWIST1
PIM3
PAWR
H2-Q4
MEOX2
SOSTDC1
H1F2
SKA1
GMIP
IGFBP7
TMEM258
GADD45A
ARSI
LUM

WTIP
BTG1
CARD11
ERMARD
SH3TC1
H2-AA
PLXNB2
MICALL2
KIF15
RPS27L
LPAR3
HSPB6
WNT4
SMAD6
DNA2
ACAA2
C1QA
RAP2B
C1QC
C1QB
UNC93B1
TTC7
NFKBID
PML
NXT1
WDR62
CAVIN3
LRG1
MGARP
KRT80
ISLR
SPRY1
FGF2
SPRED3
ITIH2
GALNT6
LDLRAP1
TACC3
TRAF3IP3
TAP1
H2-M1
MAP4K1
CHST7
CCN3
ICAM1
HTRA4
SERPINE1
VGF
CEP85
ARID5A
KLF10
TEDC1
DTL

DLGAP5
H2-DMB2
PLEKHG2
RPS16
WDHD1
GCH1
TNFRSF23
ATOH8
CLDN11
CDKN3
SLC25A43
ZBTB42
H2-DMA
GPR160
ASPG
TECTA
CKAP2
THEMIS2
EEF1A1
PTGER2
SLC16A9
RPL10A
CTNNA1
TGM2
IFI206
EGR2
TMEM156
DDX60
CCR7
CARD19
RFLNA
NINJ1
ITPRID1
RMI2
RARA
SOCS1
SMIM3
CSF3
HSPB2
CD84
PLEKHG6
SLAMF7
SCARF1
TLCD2
F11R
ASIC3
NOP2
CAMP
LASP1
TTK
RRAS
TXNIP
PMEPA1

FOXF2
EGR1
SPSB2
POLR2L
GDF15
C1S1
C1RL
ATF5
AKAP12
COLEC10
ATP6V0A4
MCL1
CTSS
POLD1
RNH1
ELOVL3
VILL
STAP2
GNGT2
GCNT1
BAIAP2L1
RPL12
CCDC68
3930402G23RIK
PRC1
CFAP157
TXNDC5
TLR4
EME1
ANPEP
F13A1
ADGRG6
RUNX2
AKNA
DAP
FANCI
ORM1
METRNL
STX11
SEC24D
ISG20
FNDC3B
IGFBP2
RNF122
HLX
NEIL3
PRSS23
PPL
ZDHHC2
PRRX2
DSE
CDSN
MOCOS

VASN
MRPS6
SDK1
BATF2
EXO1
XKR5
ARHGAP29
SLC44A3
LRRC17
TMEM120A
FGL2
EVA1C
SPSB1
MRAP
HIP1
ZC3H12D
SINHCAF
TIMELESS
SAA3
STAT2
BUB1B
THBS1
TAX1BP3
PCLAF
EMP3
CDK6
NDUFA4L2
DDX58
BTBD16
CCHCR1
SLC25A24
FAM102B
ARHGAP9
CACNG5
PPP1CA
PLEKHA4
PPP1R15A
MYBBP1A
BLVRB
PVR
TLR8
MILR1
C3AR1
GSTM2
APOC1
APOBEC1
TMEM51
DOK4
DNPH1
CLCF1
HMGN1
COL16A1
LIMD2

SH3PXD2B
KLC3
PPP1R13L
CD53
LAT2
CHIL3
SZRD1
ERF
RPS19
PIF1
FZD7
OSTC
NBL1
PRKD2
THOC6
INHBA
BDKRB1
SSR2
MYZAP
CLDN5
MDFIC
BC055324
CCNB1
MMRN2
SYT15
SERPINA3G
PIEZO2
KIF14
IRF8
HSPB8
INAVA
TMEM273
KIRREL
TSPO
TMEM45B
TREM3
TRAM2
OASL1
SYTL3
RHOD
MCM3
S100A10
SPIDR
2010003K11RIK
HNRNPF
VWA1
CMKLR1
AMMECR1
LYN
TREM1
STAB1
S100A14
S100A13

IKBKE
GJB5
GJB3
FAM83G
ESM1
DCTPP1
MUSTN1
CLSPN
MAP2K3OS
STK40
MAFF
SHC1
GUCY2C
ARRDC4
ZC3H12A
NPL
STN1
ID1
APOBR
MUC1
FOXF1
GPR151
SNAI1
SERPINB6B
MAMSTR
UPK3B
KLHL6
PTGIR
FAM89A
SYNPO
TUBA1C
HIC1
MOB1A
ARL11
IFI209
TRIM56
OTUD1
HILPDA
GIMAP5
FBXL7
MMP3
B430306N03RIK
BICDL2
PRR33
CLEC4A3
TAF10
S1PR2
ZFP469
NCMAP
A530064D06RIK
ORAI3
MGAT2
ADGRD1

C130050O18RIK
IL36G
ARF6
CDC25C
IL20RB
CTLA2A
CDKN2A
GPR4
ACKR3
ADIG
GM9493
RIN3
TENT5C
TNFAIP8L1
LNCPINT
RPS2
RASIP1
TLR7
ZBTB7C
PHF11A
ZFP36
CD300C2
SHB
TLR1
PRSS22
RPSA-PS2
RPL18A
TUBB2B
AI467606
CENPH
CENPE
TNFRSF26
TMEM88
CXCR4
OLFR1033
INSM2
HCAR2
FPR1
PQLC3
PRR15
MMS22L
TSSC4
ZFP36L2
CLEC14A
MTMR11
GAPT
GLDN
E2F8
CD109
PIK3R6
PLAUR
PILRA
TRARG1

SHE
ANKLE1
RPL37A
FAM178B
RPL27A
RBP1
KCNK6
SOX18
FAM43A
ARSJ
UNC119B
LRRC8E
TICRR
OLFML2A
GJB4
HMGA1
FOXC2
BST2
NXPH3
RPL14-PS1
CDC42SE1
KCTD11
GPRC5A
FAM83H
RHOJ
PPP1R3B
MPEG1
CKAP4
FBL
IRGM1
LTB4R1
MYCT1
GM5637
TMEM198B
PBP2
TICAM1
CD24A
NEURL3
PTGS1
GAP43
SFN
KCNE4
TGIF1
FAM180A
ERFE
ADAMTS12
MIS18BP1
NXPE5
RPS8
GM15501
RPSA-PS10
WBP1L
CD300LF

CCDC88B
CDCA4
GIMAP6
A4GALT
KLK9
MARCKSL1
STBD1
PIRT
AHCYL
ENTPD1
COL6A3
CRACR2B
SYNGR2
FRMD6
CKAP2L
LHFP
OSR1
BDNF
DEPP1
SP8
TMEM252
MYOF
GM6377
A730049H05RIK
TPCN2
FNDC9
RPL29
P2RY6
SLC35E4
ARHGAP30
CDCA2
GPHB5
CLEC4A1
CCR2
AGTR1A
C5AR1
LIX1L
KRT8
OGFR
PROKR1
DTX3L
RPS23
TMEM202
TSKU
CCDC69
TIFAB
ORAI1
PHETA2
UCN2
PRSS46
MMP12
RPL36AL
TMSB4X

CRH
NLRC3
RASD1
2610318N02RIK
SLC35C1
H2AX
PLEKHF2
LRRC25
SHISA3
AMZ1
KLK6
FAM110D
HASPIN
BPIFC
LSM10
EVA1B
GJA4
FZD2
FOXC1
LGALS3
SPRR1A
CH25H
C77080
TCF19
FAM167B
PROKR2
MMP13
RPS27RT
UBALD2
FTL1
VAMP8
PTGES
TRIM15
TMEM37
RTN4RL2
SELENON
FHIP1A
P4HA3
GIMAP9
GPR183
ERCC6L
SIX1
KIF18B
CD14
SPN
TLR6
WDFY4
ARHGEF39
RPS19BP1
ZFP579
OTOP1
AU021092
RINL

WFDC21
GYPA
ZFP217
DOCK8
RGS14
RASAL3
CD177
FPR2
HBB-B1
HBB-BS
SLC39A1
LRRC15
NRROS
MOGAT2
EZR
SMIM30
PMP2
RAP1B
JUN
RAB7B
TRIM30B
GM38525
A630001G21RIK
OAS1A
JUNB
GLIS3
CYP2F2
GPR141
YAP1
SOCS3
FES
BCL3
PRX
RAET1E
SEC61B
TARM1
TG
LGALS7
3110082I17RIK
SMAGP
IER2
SH3PXD2A
DPEP2
PTRH1
KRT16
GRWD1
LIPG
SLC39A8
CNN3
NDST1
PKP3
IIGP1
IFI205

ALKAL2
ARFGAP3
KCNN4
GIMAP4
SH3BP2
ADAM12
PLA2R1
MMRN1
EMCN
HMGB2
ACTN4
DSP
AFP
PDLIM1
KLF2
C1RA
FOXD2
ADH7
TEAD1
FBXO6
PPRC1
CDCA7
RRAS2
TCF7L1
TAFA3
TMEM150A
CLDN23
NOD2
CLCA3A1
S100A8
S100A9
EIF3B
TICAM2
NPM3
TCIM
CD248
TMEM154
CEBPB
RAB31
PTAFR
MPZ
PPP1R14B
PRELID2
IER5
CAPG
NFIL3
HMGA2
GLIPR1
SIPA1
GM7536
FXYD3
GJA5
SCIMP

TMEM140
AB124611
RPL38
APOL9A
PHEX
SAA2
ECE1
LMCD1
RPS18-PS3
LEPR
PRTN3
GM6169
BAK1
RPL32
RPL36
UNC13D
SEPTIN11
ADTRP
PALLD
GM10036
NFAM1
TPM3-RS7
GM5431
SERPINA3K
ESPL1
DOCK1
KRT6A
GPR182
CXCL2
GM8369
RPL23A
GM46442
RPL5
TMOD3
RPL28-PS1
GDA
FCER1G
OSM
NFE2
PIRB
RPL18
FCGR4
IFITM6
SEPTIN9
RPL11
LOC115489947
SLC14A1
FXYD2
ACTG2
PTK2B
B3GNT8
FCGR3
TOR4A

REG1
STFA2L1
FLOT1
RPS26-PS1
NRM
EIF4A1
KPRP
IRAK4
TMEM40
ADAMTS14
NPTX2
ALOX5AP
TPT1
ATP8B4
GM10076
SERPINB6A
RPS10-PS1
KNG2
ADGRG3
XCR1
TOR3A
H2-EB1
IFITM2
LAYN
GMFG
B2M
TRMT61A
DMKN
PRCP
CYP4F39
BLNK
FNIP2
H2-K1
GM9104
RPS7
ZIC2
CFD
RPS17
RUFY4
MYL1
GM6311
SPHK1
PPP4R1
RPS12
RPL34
RPL34-PS1
PGGHG
CD200R4
GM5088
TNFAIP8
CKS2
NECTIN2
ERBB2

T
T2
CYTL1
SERPINB2
FTL1-PS2
FTL1-PS1
RPL9-PS6
NSL1
FABP4
CNR2
KCNJ15
2310039H08RIK
RPL7A
CAV3
GM16477
ACTG1
IMPDH2
KDR
CPED1
RPL35
MSLN
SOX7
CD300LB
LDHA
GPR84
PARP10
HACD1
RPL27
GM10131
CYP26B1
RPS15
GM10132
CCDC102A
SOX11
GM8730
TNFRSF11B
S100A7A
CDC42EP5
RNF183
GPX1
CREM
KLK8
SCGB3A1
FBLN2
VRK2
HCST
RAB44
IZUMO1
ANO6
IFI27
DHRS3
CLDN19
GM12918

SERPINA3C
RPS13-PS1
SERPINA3F
GM5786
PILRB2
PILRB1
OAS1G
RPS12-PS3
GM7027
H2-M9
H2-T23
NIPAL1
H2-Q10
RPLP0
RPS28
S1PR3
NMS
LPAR5
GM14648
CENPM
CCDC134
CST7
COL8A1
LGALS1
GM11808
PHF11D
APOL9B
DOK1
GML
RPL34-PS1
GNG5
MYADM
GM4841
FLNC
PSRC1
IL3RA
COL28A1
PRDM6
GM4950
LYZ2
GM10275
WFDC17
SLFN9
NLRP1A
AHNAK
SLC47A2
IRGM2
SPDL1
HBA-A2
HBA-A1
SP140
SP110
SNORA57

GMPPB
MMP27
RNF213
GM10288
CCND1
ITGAD
HS3ST3B1
IL18BP
SERPINH1
TLNRD1
FCRLB
PEG12
NTN5
SRARP
RUNX3
HMGN2-PS
RBM47
TRABD2B
IL1RL2
DNAJC25
HRCT1
IMPDH2-PS
RPL7A-PS5
TREML2
NR2C2AP
CCDC78
NAIP5
MFSD4B3-PS
TRAPPC3L
PSMG4
GM10335
NT5DC2
STFA1
CEBPD
EEF1G
LRRN4CL
CSF2RB
CSF2RB2
NCF4
SMPD5
CCNF
ANG
TRIM6
SLFN2
SLFN10-PS
RPL37RT
G530011O06RIK
SERPINA1E
SOD3
OIP5
VAMP5
GM5127
1810058I24RIK

TRIM10
H2-D1
C4B
H2-AB1
H2-KE6
IFI204
IFI207
IFI213
GM4951
ECSCR
RPL27-PS3
RPL31
BTBD19
GM10575
CDKN2B
HBB-BT
OMP
OSGIN1
SAA1
RPL13A
NLRC5
KRTDAP
ADH1
C5AR2
EHD2
PIRA11
PIRA6
PIRA7
PIRA12
S100A16
SPC24
MEX3A
MAFB
FOXS1
SIRPB1C
THBD
SLC4A11
MORRBID
ITPRIPL1
IFIT3
CHST14
HEG1
ERICH2
SP5
RPRM
4930594M22RIK
LY6A
MSRB1
DIO3
SOX4
TRBC2
IGKV12-89
F630028O10RIK

RPL23A-PS3
PSME2B
DNM3OS
GM2000
HMGA1B
GM2606
FOXD1
SF3B5
RAET1A
RAET1B
RAET1C
RAET1D
ZFP268
AUNIP
TRIM30C
SLFN1
9530053A07RIK
GM5150
EIF5A
IGTP
IFI47
TGTP1
NAIP6
NAIP2
ARHGAP8
SERPINA3M
SERPINA3I
IFI27L2A
LY6C1
C7
KDELR2
PSME2
CCR5
XIRP1
TMEM233
CLEC7A
ACKR4
MS4A6C
RPL36A
ST6GALNAC4
B3GNT7
TMSB10
H2-DMB1
KIFC1
CSTDC4
TM4SF19
RPL39
ULBP1
RPSA-PS12
GM12174
RPL10-PS1
GM15427
H3C15

RPS15A-PS7
RPL19-PS11
GM13680
RPS6-PS4
PIRA1
RPS2-PS13
RPL17-PS5
BTC
GM12366
RPSA-PS9
GM11249
GM14586
GM13436
GM11478
GM6136
GM15710
ESRP2
RPL10A-PS1
CROCC2
GM13415
MIR22HG
SNHG15
WINCR1
FOXD2OS
4732490B19RIK
KCNMB4OS2
5330429C05RIK
GM14273
CD63-PS
GM12530
BACH2OS
XIST
CD101
MEXIS
2410006H16RIK
GM13889
AI662270
GM11714
MROH3
GM16174
GALNT2
IER5L
GM16580
SLC5A3
GM16184
BCL2A1B
PIRA2
GIMAP1
SRPX
UGT1A7C
POU3F1
GM10177
UBA52

D730045A05RIK
4930523C07RIK
RPS11-PS1
GYPC
GM6576
APOLD1
RPS27
MYL6
RPS13
HSPA1B
CCDC71L
LRRC32
SIGLEC15
RPL6L
GM6682
COL6A5
GM10269
LSM5
PHF11B
RPL36-PS12
RPS2-PS10
D030025P21RIK
1110038B12RIK
SERPINB10
RPL41
CCL21A
CSTA3
TMEM95
RPL9-PS4
SIRPB1B
ITPRIPL2
RPS2-PS6
A630023A22RIK
RPS7-PS3
GM21188
CSTA2
DYNLT1F
SIRPB1A
LHX8
PSMB9
RPS19-PS6
SNHG18
A230028O05RIK
PTGS2OS
FOXL1
2700038G22RIK
GM26519
TNFSF12
5830432E09RIK
PTGES3L
4833412C05RIK
A930007I19RIK
BIN2

SNHG6
LOCKD
MIR6236
MS4A14
GM8451
H3C4
RPS10-PS2
BCL2A1D
GM8307
MS4A4A
1600010M07RIK
BCL2A1A
LY6A2
RPSA-PS1
SH2D1B1
PCDHGC5
PCDHGC3
GM4610
RPS4X-PS
RPL36A-PS2
MIR703
AI506816
GM4332
KIF19B
GM4754
MRPL33
GM4366
GM42788
GM6794
GM36582
GM7932
SNHG1
SAMD4B
NUP62
GM5905
GM19935
GM39822
RPL10-PS5
GM5425
LILRB4B
GM30539
LILRB4A
4933412E12RIK
4930471E19RIK
GM6093
RPS18-PS5
GM19951
GM32635
DIO3OS
5033406O09RIK
GM9616
GM7143
GM36161

9330188P03RIK
EEF1AKMT4
PNP
GM19510
GM9732
GM5854
GM18787
D730005E14RIK
GM3435
E330032C10RIK
RPSA-PS7
EEF1A1-PS1
ITPRIP
GM9895
GM34432
1700001K23RIK
TMEM179B
RN18S
RN18S-RS5
RN7SK
